# Idebenone Regulates Aβ and LPS-Induced Neurogliosis and Cognitive Function Through Inhibition of NLRP3 Inflammasome/IL-1β Axis Activation

**DOI:** 10.3389/fimmu.2022.749336

**Published:** 2022-02-10

**Authors:** Hyun-ju Lee, Jin-Hee Park, Hyang-Sook Hoe

**Affiliations:** ^1^ Department of Neural Development and Disease, Korea Brain Research Institute (KBRI), Daegu, South Korea; ^2^ Department of Brain and Cognitive Sciences, Daegu Gyeongbuk Institute of Science and Technology, Daegu, South Korea

**Keywords:** neurodegenerative diseases, cognition, neurogliosis, NLRP3 inflammasome, neuroinflammation, LPS, amyloid beta

## Abstract

Idebenone is an analogue of coenzyme Q10, an electron donor in the mitochondrial electron transport chain, and thus may function as an antioxidant to facilitate mitochondrial function. However, whether idebenone modulates LPS- and Aβ-mediated neuroinflammatory responses and cognitive function *in vivo* is unknown. The present study explored the effects of idebenone on LPS- or Aβ-mediated neuroinflammation, learning and memory and the underlying molecular mechanisms in wild-type (WT) mice and 5xFAD mice, a mouse model of Alzheimer’s disease (AD). In male and female WT mice, idebenone upregulated neuroprotective NRF2 expression, rescued LPS-induced spatial and recognition memory impairments, and reduced NLRP3 priming and subsequent neuroinflammation. Moreover, idebenone downregulated LPS-mediated neurogliosis, reactive oxygen species (ROS) levels, and mitochondrial function in BV2 microglial cells and primary astrocytes by inhibiting NLRP3 inflammasome activation. In 5xFAD mice, idebenone increased neuroprotective NRF2 expression and improved amyloid beta (Aβ)-induced cognitive dysfunction. Idebenone downregulated Aβ-mediated gliosis and proinflammatory cytokine levels in 5xFAD mice by modulating the vicious NLRP3/caspase-1/IL-1β neuroinflammation cycle. Taken together, our results suggest that idebenone targets neuroglial NLRP3 inflammasome activation and therefore may have neuroprotective effects and inhibit the pathological progression of neuroinflammation-related diseases.

## Introduction

In the normal adult brain, two types of neuroglial cells, microglia and astrocytes, regulate CNS homeostasis by synchronizing neuronal activity (1). However, under neuropathological conditions, such as lipopolysaccharide (LPS) stimulation or aberrant amyloid plaque accumulation, these neuroglial cells become inflammatory microglia and reactive astrocytes, triggering reactive oxygen species (ROS) production and NLRP3 inflammasome priming/activation (2). Specifically, upon binding of LPS to its receptor TLR4, activated NF-kB promotes NLRP3/pro-IL-1β expression, and induced JNK-1 phosphorylates NLRP3 (serine 194), thereby facilitating NLRP3 self-oligomerization and inflammasome assembly (2, 3). Amyloid β (Aβ) binds to microglial receptors, including receptor for advanced glycation end products (RAGE), and is phagocytosed, resulting in cathepsin B release followed by mitochondrial ROS production (4).

Abnormal ROS accumulation induces NLRP3 inflammasome assembly followed by activation of caspase-1 and the subsequent release of the pro-inflammatory cytokines IL-1β and IL-18 (5). Prolonged activation of the NLRP3 inflammasome induces a vicious cycle of neuroinflammation (6) and excessive release of proinflammatory mediators, exacerbating cognitive decline and the progression of neurodegenerative diseases such as Alzheimer’s disease (AD). In APP/PS1 mice, a model of AD, agents that inhibit NLRP3 improve spatial memory function/synaptic plasticity/dendritic spine number and suppress microglial activation/proinflammatory cytokine expression (7). In addition, in a mouse model of traumatic brain injury, genetic ablation of NLRP3 improves recognition memory and reduces brain IL-1β and caspase-1 levels (8). Interestingly, upregulation of nuclear factor erythroid 2-related factor 2 (Nrf2), a neuroprotective transcription factor, suppresses NLRP3, IL-1β, and IL-18 protein expression in the cerebral cortex in a rat model of middle cerebral artery occlusion (9). These observations suggest that targeting the vicious NLRP3/IL-1β axis may be a feasible strategy for treating neuroinflammation-related diseases.

Idebenone is an analogue of coenzyme Q10, an electron donor in the mitochondrial electron transport chain, and thus may function as an antioxidant to facilitate mitochondrial function. Idebenone is approved by the FDA to treat Duchenne muscular dystrophy (10) and has well-established safety. Clinical and animal studies have shown that idebenone penetrates the blood-brain barrier (11–15). In rats, idebenone protects against Aβ-mediated hippocampal neuronal loss (16). In addition, idebenone has anti-inflammatory effects in LPS-stimulated BV2 microglial cells and in animal models of Parkinson’s disease and ulcerative colitis (17, 18). Moreover, idebenone attenuates NLRP3 inflammasome activation induced by ischemia in an ischemia/reperfusion rat model (19). However, the effects of idebenone on LPS- or Aβ-associated memory deficits and the vicious gliosis/ROS/NLRP3/neuroinflammation cycle have not been fully investigated.

In this study, we examined the effects of idebenone on changes in cognitive function and neurogliosis induced by LPS and its molecular mechanism of action in wild-type (WT) and 5xFAD mice. We found that idebenone ameliorated cognitive dysfunction and glial activation/inflammatory responses in LPS-treated WT mice by inhibiting NLRP3 inflammasome activation. In addition, idebenone suppressed LPS-induced intracellular ROS production, mitochondrial membrane potential disruption, and NLRP3-associated neuroinflammation in BV2 microglial cells and primary astrocytes. In 5xFAD mice, idebenone improved short-and long-term memory and downregulated Aβ-associated micro/astrogliosis and neuroinflammatory responses by altering NLRP3 inflammasome activation. Overall, the present study reveals that idebenone has therapeutic effects on neuroinflammation-related diseases by suppressing neuroglial NLRP3 inflammasome activation.

## Materials and Methods

### Idebenone

Idebenone was purchased from Sigma-Aldrich (St. Louis, MO, USA; Cat. No. I5659). Idebenone was used at a dose of 2 μM in 1% DMSO in *in vitro* assays or intraperitoneally (i.p.) administered at 50 mg/kg or 100 mg/kg in 5% DMSO, 10% polyethylene glycol (PEG) 300 and 20% Tween 80 in *in vivo* assays.

### Animals

#### 5xFAD Mice

5xFAD transgenic male mice (MMRRC Stock 34848; B6.Cg-Tg (APPSwFlLon,PSEN1*M146L*L286V)6799Vas/Mmjax) were purchased from Jackson Laboratory (Bar Harbor, ME, USA) to investigate the effect of idebenone on Aβ-associated micro/astrogliosis and activation of the NLRP3/caspase-1/IL-1β axis. This mouse model of AD carries a transgene overexpressing three mutations in human APP (Swedish mutation K670N, M671L; Florida mutation I716V; London mutation V717I) and a transgene overexpressing two mutations in human presenilin 1 (PSEN1 M146L, L286V), both regulated by the Thy1 promoter. These five mutations are associated with familial AD. Genomic DNA extracted from the tail was used for RT-PCR detection of the human APP and PSEN genes for genotyping. Prior to behavioral tests (Y maze, novel object recognition (NOR) and Morris water maze tests) and physiological analyses (immunostaining, quantitative RT-PCR, and ELISA), 3-month-old 5xFAD mice received idebenone (100 mg/kg, i.p.) or vehicle (5% DMSO + 10% PEG + 20% Tween80) once daily for two weeks.

#### Wild-Type Mice

Three-month-old male or female C57BL6/N mice (25–30 g; Orient-Bio Company, Gyeonggi-do, Korea) were randomly assigned to experimental groups and housed 3–4 per cage under a 12:12 light-dark cycle in a pathogen-free facility with free access to food and water. To investigate the effect of idebenone on LPS-mediated gliosis and NLRP3-associated neuroinflammation, male or female WT mice were injected with idebenone (50 or 100 mg/kg, i.p.) or vehicle (5% DMSO + 10% PEG + 20% Tween80) daily for 3 or 8 days; 30 min after the final injection on day 3 or 8, LPS (10 mg/kg, i.p.) or PBS was injected. Eight hours after LPS or PBS injection, the mice were sacrificed, and immunofluorescence (IF) staining, quantitative RT-PCR or ELISA was performed. To examine effects on LPS-induced memory impairment, WT mice were injected with 100 mg/kg idebenone (i.p.) or vehicle (5% DMSO + 10% PEG + 20% Tween80) followed 30 minutes later by LPS (250 μg/kg, i.p.) or PBS administration. This treatment regimen was repeated daily for 7 consecutive days. After this treatment period, the mice were subjected to Y maze, NOR, and Morris water maze tests, and brain sections were immunostained for NRF-2.

### Behavioral Tests

#### Y Maze Test

The ability of idebenone to rescue LPS-mediated memory impairments in WT mice and to improve Aβ-induced cognitive dysfunction in 5xFAD mice was assessed by performing behavioral tests as previously described with minor modifications (20, 21). Short-term spatial memory was evaluated using the Y maze test. In this test, a single mouse was allowed to freely explore the three arms (35 cm x 7 cm x 15 cm) of the maze, which met at an angle of 120°, for 5 min. Spontaneous alternations were recorded by using a video camera and manually counted. The alternation percentage was calculated by dividing the number of alternations by the number of alternation triads.

#### Novel Object Recognition Test

To evaluate recognition memory, the novel object recognition (NOR) test was performed as previously described with minor modifications (20). In the training phase, a single mouse was allowed to explore an open-field box (40 cm x 40 cm x 25 cm) containing two identical objects for 5 min. Between trials, the box and objects were cleaned thoroughly with 70% ethanol to remove odor cues. In the testing phase, which was performed 24 h after the training phase, the mouse was placed in the same box containing one familiar object and one novel object. The locations of the two objects in the apparatus were counterbalanced among the trials. All trials were recorded, and the time of exploration was counted manually. Exploratory behavior was defined by pointing of the mouse’s nose toward an object. Object preference (%) was calculated from the exploration times for the familiar and novel objects: [Object preference (%) = T _Novel_/(T _Familiar_ + T _Novel_) × 100].

#### Morris Water Maze Test

To evaluate the effects of idebenone on spatial memory, the Morris water maze test was performed as previously described with minor modifications (21). The water maze pool was 120 cm in diameter, filled to a depth of 40 cm with nontoxic, white-colored water at 25°C, and surrounded by three external-maze cues. A hidden platform was placed 1 cm below the surface of the water in the southwest quadrant of the pool throughout the training phase (from day 1 to day 4). In the training phase, each mouse underwent three trials per day at 50-min intervals for four consecutive days. The mouse was allowed a maximum of 1 min to find the hidden platform. After the first trials on day 1, the mouse was guided to the hidden platform to learn the location of the platform based on the external cues. On day 5, the platform was removed, and the mouse was allowed to explore freely for 1 min to assess spatial reference memory. Escape latency and route were recorded and analyzed with a video camera connected to a tracking device (S-MART, Pan Lab, Barcelona, Spain).

#### Immunofluorescence Staining (IF)

Brains from 5xFAD mice and WT mice were fixed in 4% paraformaldehyde overnight at 4°C followed by 30% sucrose solution for 2 days at 4°C. Coronal slices with a thickness of 35 µm were obtained by using a cryostat (Leica CM1850, Wetzlar, Germany) and were permeabilized with PBS containing 0.2% Triton X-100 (PBST) and 10% normal goat serum for 1 h at room temperature. The sections were then immunostained with an anti-NRF2, anti-Iba-1, anti-GFAP, anti-NLRP3, anti-cleaved caspase-1, anti-IL-1β, or anti-IL-6 antibody diluted in PBST with 10% normal goat serum for 24–48 h at 4°C. The sections were washed three times in PBST, incubated with the corresponding secondary antibody at room temperature for 2 h, washed three times with PBS, and mounted in antifade mounting medium with DAPI (Vector Laboratories, Burlingame, CA, USA). Fluorescence microscopy was used to acquire images of the sections (DMi8, Leica Microsystems, Wetzlar, Germany), which were analyzed by the software ImageJ (version 1.53a, National Institutes of Health, Bethesda, MD, USA). The primary and secondary antibodies are detailed in **Table 1**.

**Table 1:** List of antibodies used in this study.

Primary antibodies					
Immunogen	Host species	Dilution	Manufacturer	Catalog no.	Application
NRF2	Rabbit	1:200	Cell Signaling	12721	IF
Iba-1	Rabbit	1:500	Wako	019-19741	IF
GFAP	Rabbit	1:500	Neuromics	RA22101	IF
NLRP3	Goat	1:100	Abcam	AB4207	IF, ICC
NLRP3	Rabbit	1:500	Cell Signaling	15101	WB
β-actin	Mouse	1:1000	Santa Cruz	SC-47778	WB
Caspase-1	Rabbit	1:100	Cell Signaling	89332	IF
IL-1β	Rabbit	1:200	Abcam	AB9722	IF
IL-6	Mouse	1:100	Santa Cruz	SC-57315	IF
CD11b	Rat	1:200	Abcam	AB8878	ICC
GFAP	Chicken	1:500	Millipore	AB5541	ICC
p-STAT3^S727^	Rabbit	1:200	Abcam	AB86340	ICC, WB
p-NF-kB^S536^	Rabbit	1:200	Cell Signaling	3033	ICC
NF-kB-p65	Rabbit	1:500	Cell Signaling	8482	WB
PCNA	Mouse	1:1000	Santa Cruz	SC-56	WB
**Secondary antibodies**					
**Antibody**		**Dilution**	**Manufacturer**	**Catalog no.**	**Application**
Goat anti-rabbit IgG, 555		1:200	Invitrogen	A21428	IF, ICC
Goat anti-rabbit IgG, 488		1:200	Invitrogen	A11008	IF
Goat anti-mouse IgG, 488		1:200	Invitrogen	A11001	IF
Donkey anti-goat, 555		1:200	Invitrogen	A21432	IF
Goat anti-chicken IgG, 488		1:200	Abcam	A150169	IF
Goat anti-rat IgG, FITC		1:200	Invitrogen	A18866	ICC
Goat anti-rabbit, HRP		1:5000	Enzo	ADI-SAB-300-J	WB
Goat anti-mouse, HRP		1:5000	Enzo	ADI-SAB-100-J	WB

#### ELISA

The effects of idebenone on cortical and hippocampal levels of IL-1β, a major initiator of inflammation induced by NLRP3 inflammasome activation, were assessed in male and female WT and 5xFAD mice. WT mice were injected with 100 mg/kg idebenone (i.p.) or vehicle (5% DMSO + 10% PEG + 20% Tween80) daily for 8 days. Thirty minutes after the final injection on day 8, LPS (10 mg/kg, i.p.) or PBS was administered, and 8 h later, cortical and hippocampal tissues were dissected and stored at −80°C until analysis. 5xFAD mice were injected with 100 mg/kg idebenone (i.p.) or vehicle daily for 14 days, and cortical and hippocampal tissues were dissected and stored at −80°C until analysis. To obtain total protein, the tissue was homogenized in RIPA buffer with protease inhibitor and phosphatase inhibitor and centrifuged at 10,000 rpm and 4°C for 15 min, and the supernatant was collected. Total protein concentrations were determined *via* BCA protein assays. Cortical and hippocampal IL-1β levels were measured using an ELISA kit (Cat. no. 88-7013-88, Invitrogen, Waltham, MA, USA) as described by the manufacturer.

### BV2 Microglial Cells and Mouse Primary Astrocytes

#### BV2 Microglial Cells

The effects of idebenone on LPS-mediated ROS/NLRP3/IL-1β axis activation were investigated in BV2 microglial cells (a generous gift from Dr. Kyung-Ho Suk). High-glucose DMEM (Invitrogen, Carlsbad, CA, USA) was used as the culture medium and was supplemented with 5% fetal bovine serum (FBS, Invitrogen, Carlsbad, CA, USA), 100 units/ml penicillin, and 100 μg/ml streptomycin. A 5% CO_2_ incubator was used to maintain the cells. For each assay, BV2 microglial cells (2.0x10^4^/well for ICC and 2.5x10^5^/well for all other experiments) were treated with idebenone (2 μM) or vehicle (1% DMSO) for 30 min followed by LPS (1 μg/ml) or PBS for 5.5 h (for nuclear fractionation and ICC) or 23.5 h (for all other experiments).

#### Mouse Primary Astrocytes

Primary astrocytes were prepared as previously described (22). In brief, mixed cells obtained from postnatal day (P1) C57BL/6 mouse whole brain tissues minced through 70-μm nylon mesh were cultured in a 5% CO_2_ incubator in low-glucose DMEM (1,000 mg/l glucose) with 10% FBS, 100 unit/ml penicillin, and 100 μg/ml streptomycin. Primary microglial cells were detached on day 14 by shaking overnight at 250 rpm and RT, and primary astrocytes were subsequently dissociated using trypsin-EDTA. After centrifugation at 2000 rpm for 30 min, the pellet (primary astrocytes) was used for seeding experiments. For each assay, primary astrocytes (5.0x10^4^/well for ICC and 7.0x10^5^/well for all other experiments) were treated with idebenone (2 μM) or vehicle (1% DMSO) for 30 min followed by LPS (200 ng/ml) or PBS for 5.5 h (for ICC of p-NF-kB and p-STAT3) or 23.5 h (for all other experiments).

#### Immunocytochemistry (ICC)

To assess the effect of idebenone on LPS-evoked NLRP3 activation *in vitro*, BV2 microglial cells or mouse primary astrocytes were seeded on glass cover slides and treated as described in the previous section. After fixing in 4% paraformaldehyde containing 0.4% (w/v) sucrose for 10 min, the cells were incubated with an anti-NLRP3 antibody diluted in GDB buffer (0.1% gelatin, 0.3% Triton X-100, 16 mM sodium phosphate, pH 7.4, and 450 mM NaCl) overnight at 4°C. The cells were then washed three times with PBS, incubated with the corresponding secondary antibody at room temperature for 2 h, and washed again with PBS. After incubation in DAPI solution (1:1000 in PBS with 0.01% DMSO, Invitrogen, Carlsbad, CA, USA), the cells were mounted in fluorescence mounting medium (DAKO, Santa Clara, CA, USA). To identify the underlying signaling mechanism by which idebenone regulates the LPS-induced NLRP3 inflammatory pathway, BV2 microglial cells or mouse primary astrocytes were treated as described in the previous section. Then, ICC was conducted with an anti-p-NF-kB^S536^ or anti-p-STAT3^S727^ antibody and corresponding secondary antibody. The primary and secondary antibodies used for ICC are detailed in **Table 1**. Images acquired with a DMi8 inverted fluorescence microscope (Leica Microsystems, Wetzlar, Germany) were analyzed using ImageJ (version 1.53a, US National Institutes of Health, Bethesda, MD, USA).

#### Real-Time Quantitative PCR (q-PCR)

To investigate whether idebenone regulates LPS-mediated NLRP3 inflammasome activation and subsequent proinflammatory cytokine production or Aβ-associated NLRP3 inflammasome upregulation, BV2 microglial cells, primary astrocytes, WT mice and 5xFAD mice were treated as described above. Total RNA was extracted from BV2 microglial cells, primary astrocytes, or brain tissue (cortex and hippocampus) using TRIzol (Invitrogen, Waltham, MA, USA) according to the manufacturer’s instructions. cDNA was synthesized by reverse transcription of the total RNA (1 μg) using the Superscript cDNA Premix Kit II with oligo (dT) primers (GeNetBio, Chungman, Korea) and used with the primers listed in **Table 2** in real-time qPCR with Fast SYBR Green Master Mix (Thermo Fisher Scientific, Waltham, MA, USA) in a QuantStudio 5 Real-Time PCR System (Applied Biosystems, Thermo Fisher Scientific, Waltham, MA, USA). The fold change relative to the vehicle-treated control was calculated after normalizing the cycle threshold (Ct) values to the value for *Gapdh*.

**Table 2:** Sequences of primers used for real-time qPCR.

Gene		Sequence
*nlrp3*	Forward	5’-TCC ACA ATT CTG ACC CAC AA-3’
	Reverse	5’-ACC TCA CAG AGG GTC ACC AC-3’
*pro-il-1β*	Forward	5’- TCT TTG AAG TTG ACG GAC CC -3’
	Reverse	5’- TGA GTG ATA CTG CCT GCC TG -3’
*il-1β*	Forward	5’-TTG ACG GAC CCC AAA AGA TG-3’
	Reverse	5’-AGG ACA GCC CAG GTC AAA G -3’
*tnf-α*	Forward	5’- TCC AGG CGG TGC CTA TGT -3’
	Reverse	5’- GCC CCT GCC ACA AGC A -3’
*il-6*	Forward	5’-CCA CGG CCT TCC CTA CTT C-3’
	Reverse	5’-TTG GGA GTG GTA TCC TCT GTG A-3’
*cox-2*	Forward	5’-CCA CTT CAA GGG AGT CTG GA -3’
	Reverse	5’-AGT CAT CTG CTA CGG GAG GA-3’
*gapdh*	Forward	5’-TGG GCT ACA CTG AGG ACC ACT-3’
	Reverse	5’-GGG AGT GTC TGT TGA AGT CG-3’

#### Intracellular Reactive Oxygen Species (ROS) Measurement

To investigate the ability of idebenone to modulate LPS-evoked intracellular ROS levels, BV2 microglial cells or mouse primary astrocytes were treated as described above and subsequently incubated with 10 μM dichlorodihydrofluorescein diacetate (DCFH-DA, Invitrogen, catalog number: D399, Waltham, MA, USA) for 40 min at 37°C. After washing the cells twice with PBS, the fluorescence intensity (excitation 485 nm, emission 535 nm) was measured and analyzed by using a FlexStation 3 Multi-mode Microplate Reader (Molecular Devices, Jan Jose, CA, USA).

#### Mitochondrial Membrane Potential (△ΨM) Analysis

To assess the ability of idebenone pretreatment to attenuate LPS-induced mitochondrial membrane potential disruption *in vitro*, BV2 microglial cells or mouse primary astrocytes were treated as described above. The BV2 microglial cells were subsequently incubated with 1 μg/ml JC-1 fluorescent cationic probe (Invitrogen, catalog number: T3168, Waltham, MA, USA) for 20 min at 37°C, whereas the primary astrocytes were incubated with 5 μg/ml JC-1 for 40 min at 37°C. After washing with PBS, the intensities of the red fluorescence of JC-1 aggregates (excitation at 554 nm, emission at 590 nm) and green fluorescence of JC-1 monomers (excitation at 485 nm, emission at 535 nm) in the cells were measured by using a FlexStation 3 Multi-mode Microplate Reader (Molecular Devices, San Jose, CA, USA). Finally, the ratio of red signal to green signal was calculated to analyze the mitochondrial membrane potential.

#### Western Blot

To examine whether idebenone pretreatment ameliorates LPS-induced NLRP3 upregulation *in vitro*, BV2 microglial cells or mouse primary astrocytes were treated as described above, followed by western blotting with anti-NLRP3 and β-actin antibodies. In brief, the treated cells were lysed on ice for 5 min in lysis buffer (50mM Tris, pH 7.4, 1% Triton X-100, 2mM CaCl_2,_ and 2mM MgCl_2_) containing protease inhibitor and centrifuged at 12000 rpm for 15 min. The protein concentration in the supernatant was then determined using protein assay reagents (Bio-Rad Laboratories, Hercules, CA, USA). An aliquot of lysate equivalent to 15 μg of protein was boiled at 100°C for 5 min, separated on an 8% SDS-polyacrylamide gel, and electrotransferred to a polyvinylidene difluoride (PVDF) membrane (Millipore, Bedford, MA, USA). The PVDF membrane was blocked with 5% skim milk in TBST and incubated with the primary antibody overnight at 4°C followed by the horseradish peroxidase-conjugated secondary antibody for 1 h. Finally, detection was realized using ECL solution (ATTO, Tokyo, Japan), and images were obtained and analyzed using Fusion Capt Advance software (Vilber Lourmat, Eberhardzell, Germany).

To verify the signaling pathways underlying NLRP3 inflammasome regulation, BV2 microglial cells were treated as described above and subjected to western blotting with anti-p-STAT3^S727^, anti-NF-kB-p60, and anti-PCNA antibodies. The cells were lysed on ice for 5 min in cytosolic fractionation buffer (10 mM HEPES, pH 8.0, 1.5 mM MgCl_2_, 10 mM KCl, 0.5 mM DTT, 300 mM sucrose, 0.1% NP-40, and 0.5 mM PMSF) containing protease and phosphatase inhibitors. The pellet obtained after centrifugation for 1 min at 10,000 rpm and 4°C was further lysed on ice for 15 min in nuclear fractionation buffer (10 mM HEPES pH 8.0, 20% glycerol, 100 mM KCl, 100 mM NaCl, 0.2 mM EDTA, 0.5 mM DTT, and 0.5 mM PMSF) containing protease and phosphatase inhibitors. After centrifugation again for 15 min at 10,000 rpm and 4°C, the supernatant was collected and analyzed by western blot as described above.

#### siRNA Transfection

To verify whether idebenone regulates LPS-induced inflammatory responses in an NLRP3-dependent manner, BV2 microglial cells were transfected with small interfering RNA (siRNA) targeting mouse NLRP3 (Santa Cruz, Dallas, TX, USA). In brief, NLRP3 siRNA or scramble siRNA (30 µM) was diluted in Opti-MEM medium (Thermo Scientific, Waltham, MA, USA) and added to 1 µl of Lipofectamine^®^ RNAiMAX reagent (Thermo Scientific, Waltham, MA, USA) diluted in 49 µl of Opti-MEM medium. The siRNA complex suspension was incubated at room temperature for 40 min and added to a 24-well cell culture plate seeded with BV2 microglial cells. Twenty-four hours after transfection, the NLRP3 knockdown efficiency as analyzed, and the cells were treated with idebenone (2 μM) or vehicle (1% DMSO) for 30 min followed by LPS (1 μg/ml) or PBS for 23.5 h. The idebenone-treated cells were then prepared for q-PCR analysis to measure NLRP3 and/or proinflammatory mediator levels.

### Statistical Analysis

Graph generation and statistical analysis were conducted using GraphPad Prism 7 software (GraphPad Software, San Diego, CA, USA). Data are presented as individual data points and the mean ± SEM. Pairwise comparisons were performed using a Student’s *t*-test, while multiple comparisons were performed using one-way analysis of variance (ANOVA) with Tukey’s, Bonferroni’s, Sidak’s, Holm-Sidak’s multiple-comparisons test or Fisher’s LSD test. Asterisks indicate significance: * *p* < 0.05, **p < 0.01, and *** *p* < 0.001.

## Results

### Idebenone Rescues LPS-Mediated Memory Impairments in Male and Female Wild-Type Mice

**Figure 1:**
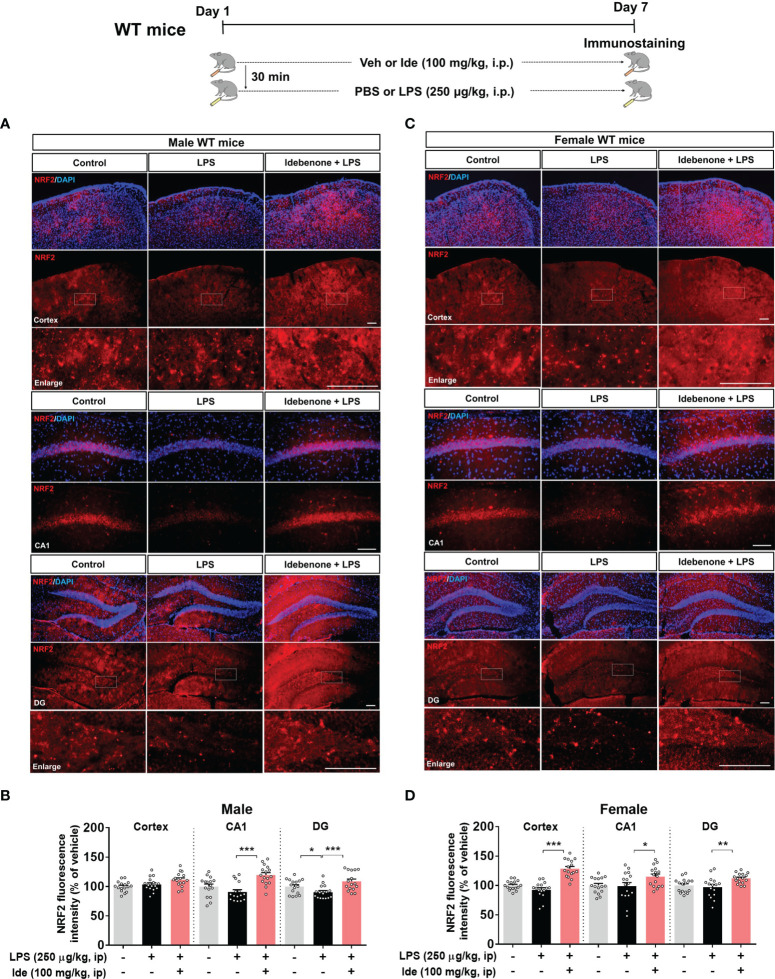
Idebenone upregulates the neuroprotective marker NRF2 in LPS-treated male and female wild-type mice. **(A, C)** Male and female wild-type mice were administered idebenone (100 mg/kg, i.p.) or vehicle (5% DMSO + 10% PEG + 20% Tween80) followed 30 min later by LPS (250 μg/kg, i.p.) or PBS administration. This regimen was repeated daily for 7 consecutive days, and immunofluorescence staining with an anti-NRF2 antibody was conducted. **(B, D)** Quantification of the fluorescence intensity of NRF2 in brain slices from LPS-treated male and female wild-type mice (n = 16 brain slices from 4 mice/group). *p < 0.05, **p < 0.01, and ***p < 0.001. Scale bar = 100 μm.

Before examining the impact of idebenone on LPS-evoked learning and memory impairments, we assessed the potential neuroprotective effects of idebenone in LPS-treated male and female WT mice. Male and female WT mice were injected with idebenone (100 mg/kg, i.p.) or vehicle (5% DMSO + 10% PEG + 20% Tween80) followed by LPS (250 μg/kg, i.p.) or PBS administration 30 min later; this regimen was repeated daily for 7 consecutive days, and immunofluorescence staining was performed with an anti-NRF2 antibody (a neuroprotective marker). NRF2 expression was increased in the CA1 and DG of idebenone-treated male WT mice compared with male WT mice injected with LPS only (**Figures 1A, B**). Idebenone-treated female WT mice also exhibited NRF2 upregulation in the cortex and hippocampus compared with female WT mice injected with LPS alone (**Figures 1C, D**). These data suggest that idebenone rescues the LPS-mediated decrease in NRF2 levels in male and female WT mice.

**Figure 2:**
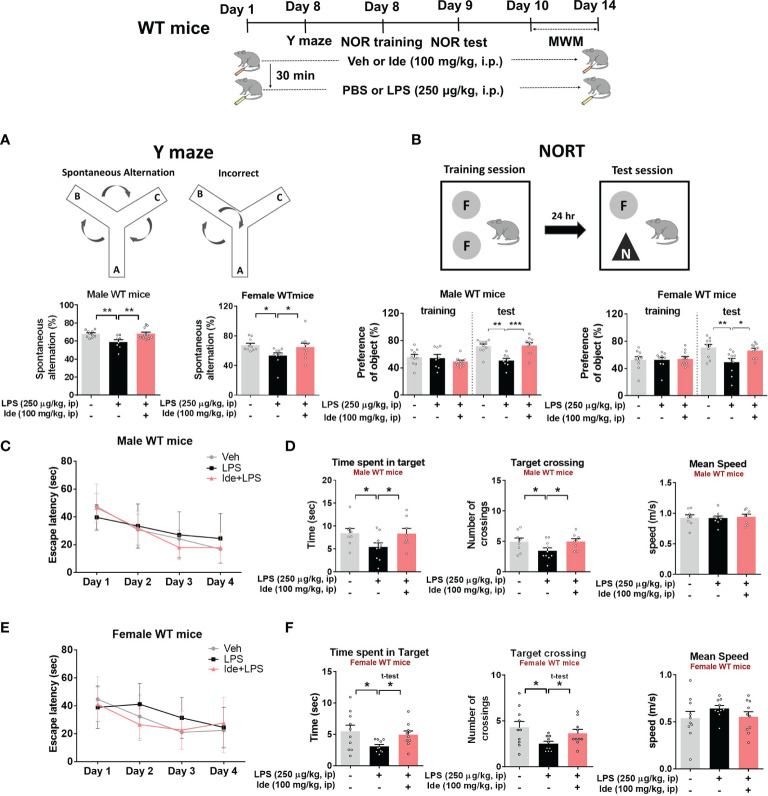
Idebenone attenuates LPS-induced memory impairments in male and female wild-type mice. Male and female wild-type mice were treated as described in **Figure 1**, and Y maze, novel object recognition (NOR), and Morris water maze tests were performed on days 8, 9, and 10–14, respectively. **(A)** Y maze diagram and quantification of spontaneous alternations (n = 8-10/group). **(B)** NOR test diagram and quantification of object preference (n = 8-10/group). **(C, E)** Escape latency to find the hidden platform during the training session of the Morris water maze test (n = 8-10/group). **(D, F)** Time spent in the target quadrant, number of target crossings, and mean speed in the Morris water maze probe test (n = 8-10/group). *p < 0.05, **p < 0.01 and ***p < 0.001.

Several studies have demonstrated that idebenone modulates inflammation, which impacts cognitive function, *in vitro* and *in vivo* (23, 24). However, whether idebenone alters LPS-mediated cognitive dysfunction is unknown. Thus, we tested the effects of idebenone on LPS-mediated learning and memory. For this experiment, WT mice were treated as described in the previous paragraph, and on days 8 to 14, Y maze, NOR, and Morris water maze tests were performed. Importantly, we found that idebenone pretreatment improved spontaneous alternations in the Y maze test compared with LPS treatment alone in both male and female WT mice (**Figure 2A**). In addition, idebenone-pretreated male and female WT mice exhibited significantly greater preference for novel objects compared with male and female WT mice treated with LPS only (**Figure 2B**). Moreover, in the Morris water maze test, idebenone-pretreated male mice spent a significantly greater time in the target area and crossed the target more frequently, whereas swimming speed was unchanged compared with male mice treated with LPS alone (**Figures 2C, D**). For female WT mice, idebenone pretreatment significantly enhanced the time spent in the target area and the number of target crossings without altering swimming speed (**Figures 2E, F**). These data suggest that idebenone restores the LPS-evoked deficits of spatial and recognition memory in WT mice.

### Idebenone Reduces LPS-Induced Microgliosis and Astrogliosis in Male and Female Wild-Type Mice

Idebenone alleviates neuroinflammation in mouse models of Parkinson’s disease (PD) and ischemia as well as inflammatory responses in mice with ulcerative colitis (17–19). Given the ability of idebenone to modulate neuroinflammatory responses, we examined whether idebenone affects LPS-mediated microgliosis and astrogliosis in WT mice. For this experiment, we chose idebenone doses of 50 and 100 mg/kg based on previous clinical and animal studies (25–28). In detail, 3-month-old male WT mice were administered idebenone (50 or 100 mg/kg, i.p.) or vehicle (5% DMSO + 10% PEG + 20% Tween80) daily for 3 days. On day 3, the injection of idebenone or vehicle was followed by injection of LPS (10 mg/kg, i.p.). Surprisingly, immunostaining of brain slices with anti-Iba-1 or anti-GFAP antibodies revealed that 3 days of daily idebenone injections had no effect on LPS-evoked microglial activation (**Supplementary Figure 1**) but significantly reduced LPS-mediated astrocytic activation in the cortex and hippocampus in male WT mice (**Supplementary Figure 2**).

**Figure 3:**
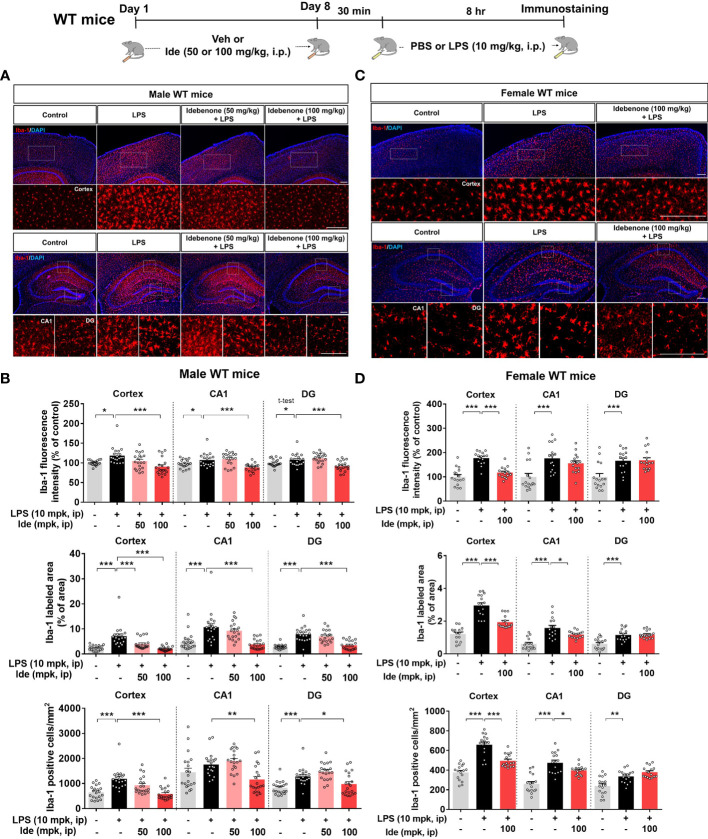
Idebenone inhibits LPS-mediated microglial activation, hypertrophy, and proliferation in male and female wild-type mice. **(A, C)** Representative image of Iba-1 immunofluorescence in the cortex and hippocampus. Idebenone (50 or 100 mg/kg, i.p.) or vehicle was administered to male and female wild-type mice daily for 8 consecutive days. Thirty minutes after the final injection, LPS (10 mg/kg, i.p.) or PBS was administered, and 8 h later, the mice were sacrificed, and brain tissue was immunostained with an anti-Iba-1 antibody. **(B, D)** Quantification of the fluorescence intensity of Iba-1; Iba-1-immunolabeled area; and number of Iba-1-positive cells (Male WT: n = 20 brain slices from 5 mice/group; Female WT: n = 16 brain slices from 4 mice/group). *p < 0.05, **p < 0.01 and ***p < 0.001. Scale bar = 200 μm.

Next, we assessed the effect of a longer duration of idebenone treatment on LPS-induced micro- and astrogliosis. For this experiment, 3-month-old male WT mice were treated as described above, except that the treatment period was 8 days instead of 3. Interestingly, this longer period of idebenone treatment significantly inhibited LPS-evoked Iba-1 intensity, Iba-1-labeled fractional area, and the number of Iba-1-positive microglial cells in a dose-dependent manner in male WT mice (**Figures 3A, B**).

**Figure 4:**
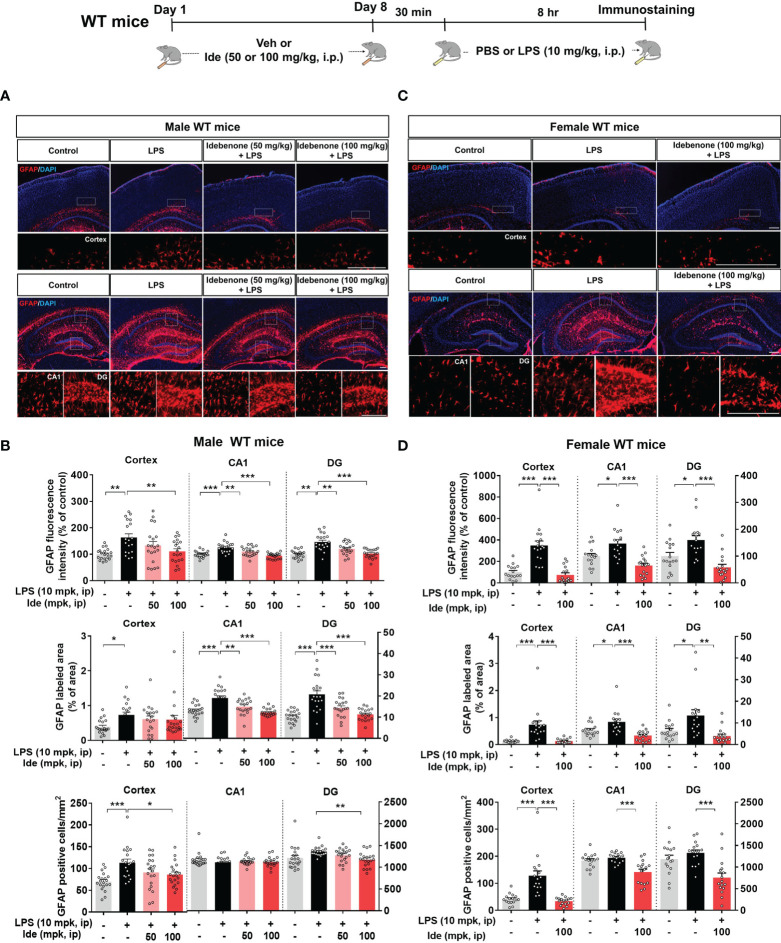
Idebenone suppresses astrogliosis and astrocytic hyperplasia and proliferation in LPS-injected male and female wild-type mice. **(A, C)** Representative images of GFAP immunofluorescence in the cortex and hippocampus. Idebenone (50 or 100 mg/kg, i.p.) or vehicle was administered to male and female wild-type mice daily for 8 consecutive days. Thirty minutes after the final injection, LPS (10 mg/kg, i.p.) or PBS was administered, and 8 h later, the mice were sacrificed, and brain tissue was immunostained with an anti-GFAP antibody. **(B, D)** Quantification of the fluorescence intensity of GFAP; GFAP-immunolabeled area; and number of GFAP-positive cells (Male WT: n = 19–20 brain slices from 5 mice/group; Female WT: n = 16–17 brain slices from 4 mice/group). *p < 0.05, **p < 0.01, and ***p < 0.001. Scale bar = 200 μm.

To determine whether idebenone differentially regulates LPS-induced microgliosis in female WT mice, 3-month-old female WT mice were treated for 8 days as described above for male WT mice. In female WT mice, idebenone significantly suppressed cortical but not hippocampal LPS-induced Iba-1 fluorescence intensity (**Figures 3C, D**). In addition, idebenone significantly reduced the LPS-evoked Iba-1-labeled fractional area and number of Iba-1-positive microglial cells in the cortex and hippocampal CA1 of female WT mice (**Figures 3C, D**). Idebenone treatment also significantly reduced LPS-mediated GFAP intensity in the cortex and hippocampus, LPS-induced GFAP-positive fractional area in the hippocampus, and the number of GFAP-labeled astrocytic cells in the cortex and hippocampal DG region in both male (**Figures 4A, B**) and female WT mice (**Figures 4C, D**). These data indicate that an idebenone dose of 100 mg/kg and a longer treatment period further reduce LPS-evoked glial activation in WT mice compared with a smaller dose or shorter treatment period. In addition, idebenone does not appear to differentially regulate LPS-induced microgliosis in female compared with male WT mice.

### Idebenone Alleviates LPS-Evoked NLRP3 Inflammasome Activation and Proinflammatory Cytokine Levels in Male and Female Wild-Type Mice

**Figure 5:**
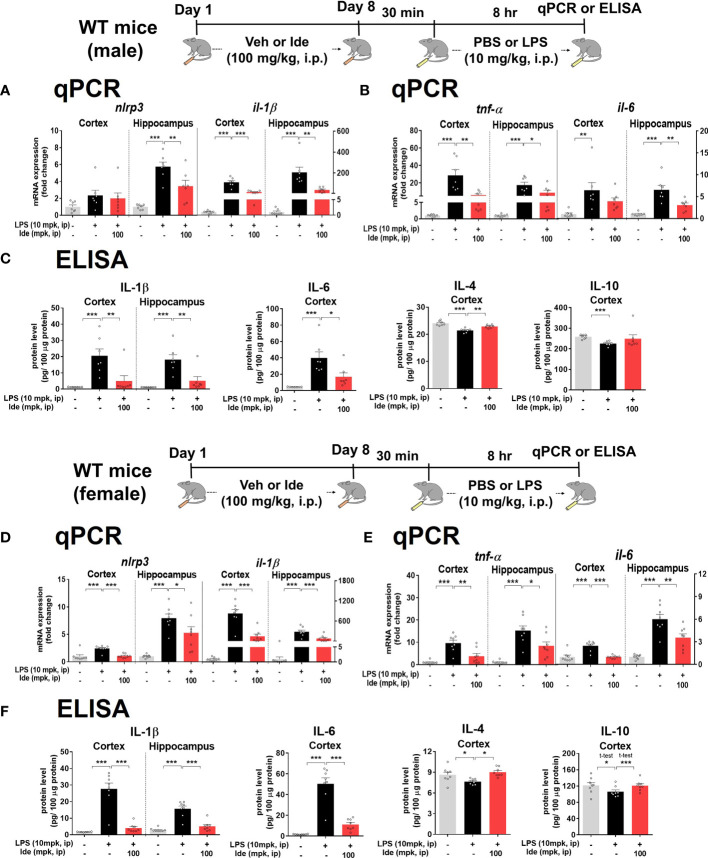
Idebenone ameliorates LPS-evoked NLRP3 inflammasome activation and neuroinflammation in male and female wild-type mice. **(A, D)** Relative mRNA levels of *nlrp3* and *il-1β* in the cortex and hippocampus. Idebenone (100 mg/kg, i.p.) or vehicle was administered to male and female wild-type mice daily for 8 consecutive days. Thirty minutes after the last injection, LPS (10 mg/kg, i.p.) or PBS was administered, and 8 h later, the cortex and hippocampus were dissected, and real-time PCR was performed (n = 7–8 mice/group). **(B, E)** Relative mRNA levels of *tnf-α* and *il-6* in the cortex and hippocampus in male and female WT mice (n = 7-8 mice/group). **(C, F)** ELISA analysis of IL-1β, IL-6, IL-4, and IL-10 protein levels in male and female WT mice (n = 7-8 mice/group). *p < 0.05, **p < 0.01, and ***p < 0.001.

Since LPS-primed NLRP3 is involved in initiating inflammatory responses (29) and idebenone was previously reported to regulate NLRP3 activation in oxygen-glucose-deprived BV2 microglial cells and PC12 cells (19), we examined the effects of idebenone on LPS-induced NLRP3 upregulation. For this experiment, male and female WT mice were treated according to the paradigms described in **Figures 3**, **4**, respectively. Importantly, LPS-activated *nlrp3* mRNA expression was significantly reduced in the hippocampus but not in the cortex in male WT mice treated with idebenone (**Figure 5A**). In addition, the increases in *il-1β* mRNA induced by LPS were remarkably suppressed in the cortex and hippocampus in idebenone-treated male WT mice (**Figure 5A**).

We then investigated whether idebenone regulates proinflammatory cytokine release mediated by NLRP3 inflammasome activation. In idebenone-treated male WT mice, LPS-induced cortical/hippocampal *tnf-α* mRNA levels were significantly reduced (**Figure 5B**). Moreover, LPS-induced *il-6* mRNA levels were significantly decreased in the hippocampus but not the cortex in idebenone-treated male WT mice (**Figure 5B**). To further confirm these findings, we conducted ELISA and found that idebenone significantly decreased the LPS-mediated increases in IL-1β and IL-6 protein levels and significantly reversed the LPS-induced downregulation of IL-4 protein levels in male WT mice (**Figure 5C**). However, no effect of idebenone treatment on LPS-induced IL-10 levels was observed.

Next, we examined whether idebenone differentially modulates LPS-primed NLRP3 levels in female WT mice. Idebenone significantly suppressed the LPS-evoked enhancement of *nlrp3* mRNA levels in the cortex and hippocampus in female WT mice (**Figure 5D**). LPS-activated cortical/hippocampal *il-1β*, *tnf-α*, and *il-6* mRNA levels were also significantly downregulated in idebenone-treated female WT mice (**Figures 5D, E**). Moreover, idebenone significantly suppressed LPS-evoked IL-1β and IL-6 protein levels and reversed the LPS-induced downregulation of IL-4 and IL-10 protein levels in the cortex and/or hippocampus in female WT mice (**Figure 5F**). These results suggest that idebenone attenuates LPS-evoked proinflammatory cytokine levels through NLRP3 inflammasome inactivation in both male and female WT mice.

### Idebenone Regulates the ROS/NLRP3/IL-1β Axis in LPS-Stimulated BV2 Microglial Cells

**Figure 6:**
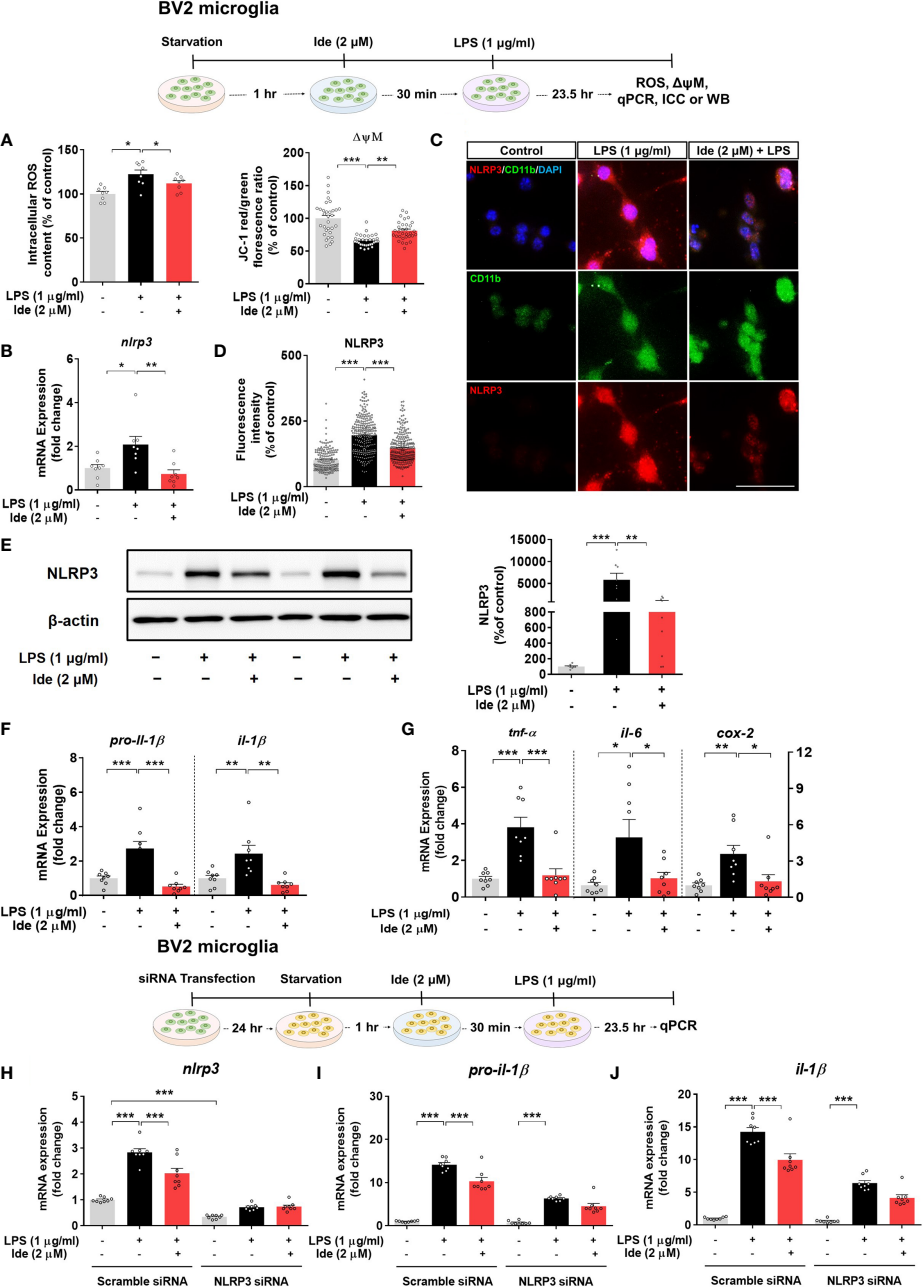
Idebenone alters LPS-evoked ROS/mitochondrial membrane potential and regulates LPS-mediated inflammatory responses in an NLRP3-dependent manner in BV2 microglial cells. Cells pretreated with idebenone (2 μM) or vehicle (1% DMSO) for 30 min were treated with LPS (1 μg/ml) or PBS for 23.5 h. ROS and mitochondrial membrane potential were then analyzed, and real-time PCR, immunocytochemistry, and western blotting were performed. **(A)** Relative intracellular ROS levels and mitochondrial membrane potential (ROS, n = 8/group; mitochondrial membrane potential, n = 32/group). **(B)** Relative *nlrp3* mRNA levels (n = 8/group). **(C, D)** Representative immunocytochemistry image and quantification of NLRP3 expression (Veh, n = 166; LPS, n = 256; and Ide + LPS, n = 267). **(E)** Representative immunoblots and quantification of NLRP3 protein levels (n = 8/group). **(F)** Relative mRNA levels of *pro-il-1β* and *il-1β* (n = 8/group). **(G)** Relative mRNA levels of *tnf-α, il-6*, and *cox-2* (n = 8/group). **(H–J)** Relative *nlrp3, pro-il-1β* and *il-1β* mRNA levels in BV2 microglial cells transfected with control (scramble) or NLRP3 siRNA and treated with idebenone followed by LPS (n = 8/group). *p < 0.05, **p < 0.01, and ***p < 0.001. Scale bar = 50 μm.

To investigate the effects of idebenone on LPS-induced neuroinflammatory responses and its molecular mechanisms of action *in vitro*, we first tested whether idebenone alters ROS production, which plays a crucial role in NLRP3 inflammasome activation (30). We found that idebenone treatment ameliorated the significant increase in intracellular ROS production induced by LPS in BV2 microglial cells (**Figure 6A**, left panel).

We then examined the effect of idebenone on mitochondrial membrane potential by using the dye JC-1. In BV2 microglial cells, LPS significantly reduced the ratio of JC-1 aggregates (red) to JC-1 monomers (green), indicating mitochondrial membrane potential disruption, but this decrease was attenuated by pretreatment with idebenone (**Figure 6A**, right panel). These results indicate that idebenone promotes mitochondrial function in BV2 microglial cells.

Next, we investigated whether idebenone modulates LPS-primed *nlrp3* mRNA and NLRP3 protein levels *in vitro* by performing real-time PCR, ICC and western blot. Pretreatment of BV2 microglial cells with idebenone significantly inhibited the increases in *nlrp3* mRNA and NLRP3 protein levels induced by LPS (**Figures 6B–E**). Related to NLRP3 downregulation, idebenone pretreatment significantly suppressed the LPS-mediated increases in *pro-il-1β*, *il-1β*, *tnf-α, il-6*, and *cox-2* mRNA levels in BV2 microglial cells (**Figures 6F, G**). These data suggest that idebenone modulates LPS-primed NLRP3 inflammasome activation and subsequent proinflammatory responses in BV2 microglial cells.

We subsequently examined whether idebenone suppresses LPS-induced proinflammatory cytokine expression in an NLRP3-dependent manner. BV2 microglial cells were transfected with siRNAs targeting NLRP3 with a silencing efficiency of 65.79% compared with the control siRNA (**Figure 6H**). Idebenone did not suppress LPS-mediated *pro-il-1β* and *il-1β* mRNA levels in NLRP3 siRNA-transfected BV2 microglial cells, implying that idebenone regulates LPS-evoked inflammatory responses in an NLRP3-dependent manner (**Figures 6I, J**).

**Figure 7:**
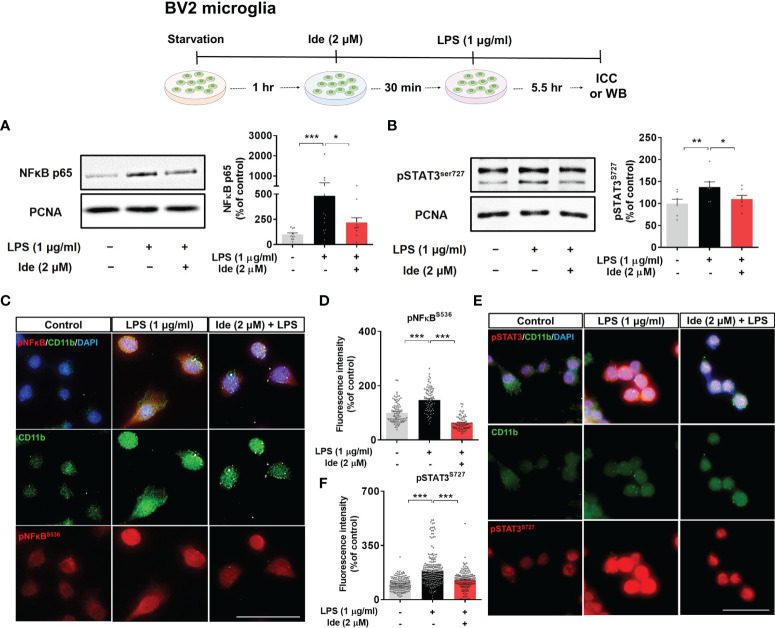
Idebenone downregulates LPS-induced NF-kB and STAT3 signaling in BV2 microglial cells. Cells pretreated with idebenone (2 μM) or vehicle (1% DMSO) for 30 min were treated with LPS (1 μg/ml) or PBS for 5.5 h, and western blotting and immunocytochemistry were performed. **(A)** Representative immunoblots and quantification of nuclear NF-kB-p65 levels (n = 11/group). **(B)** Representative immunoblots and quantification of nuclear p-STAT3 levels (n = 7/group). **(C–F)** Representative immunocytochemistry image and quantification of p-NF-kB^S536^ and p-STAT3^S727^ expression (Veh, n = 89; LPS, n = 83; and Ide + LPS, n = 76 for p-NF-kB^S536^ and Veh, n = 180; LPS, n = 253; and Ide + LPS, n = 158 for p-STAT3^S727^). *p < 0.05, **p < 0.01, and ***p < 0.001. Scale bar = 50 μm.

Next, we assessed whether idebenone regulates the transcription factor p-STAT3 and/or p-NF-kB in the nucleus to modulate LPS-induced NLRP3 inflammasome activation and proinflammatory cytokine levels. BV2 microglial cells sequentially treated for 30 min with 2 μM idebenone or 1% DMSO and for 5.5 h with 1 μg/ml LPS or PBS were subjected to subcellular fractionation. Western blotting with anti-p-STAT3^S727^ or anti-NF-kB-p65 antibodies showed that idebenone significantly downregulated nuclear p-STAT3^S727^ and NF-kB-p65 protein levels (**Figures 7A, B**). Further confirming these findings, ICC showed that idebenone significantly suppressed LPS-evoked translocation of p-STAT3^S727^ and p-NF-kB^S536^ to the nucleus (**Figures 7C–F**). Collectively, these data indicate that idebenone suppresses LPS-mediated neuroinflammatory responses by inhibiting activation of the NLRP3/IL-1β axis and NF-kB/STAT3 signaling in BV2 microglial cells.

### Idebenone Modulates LPS-Evoked ROS, Mitochondrial Membrane Potential, and NLRP3 Inflammatory Signaling in Primary Astrocytes

**Figure 8:**
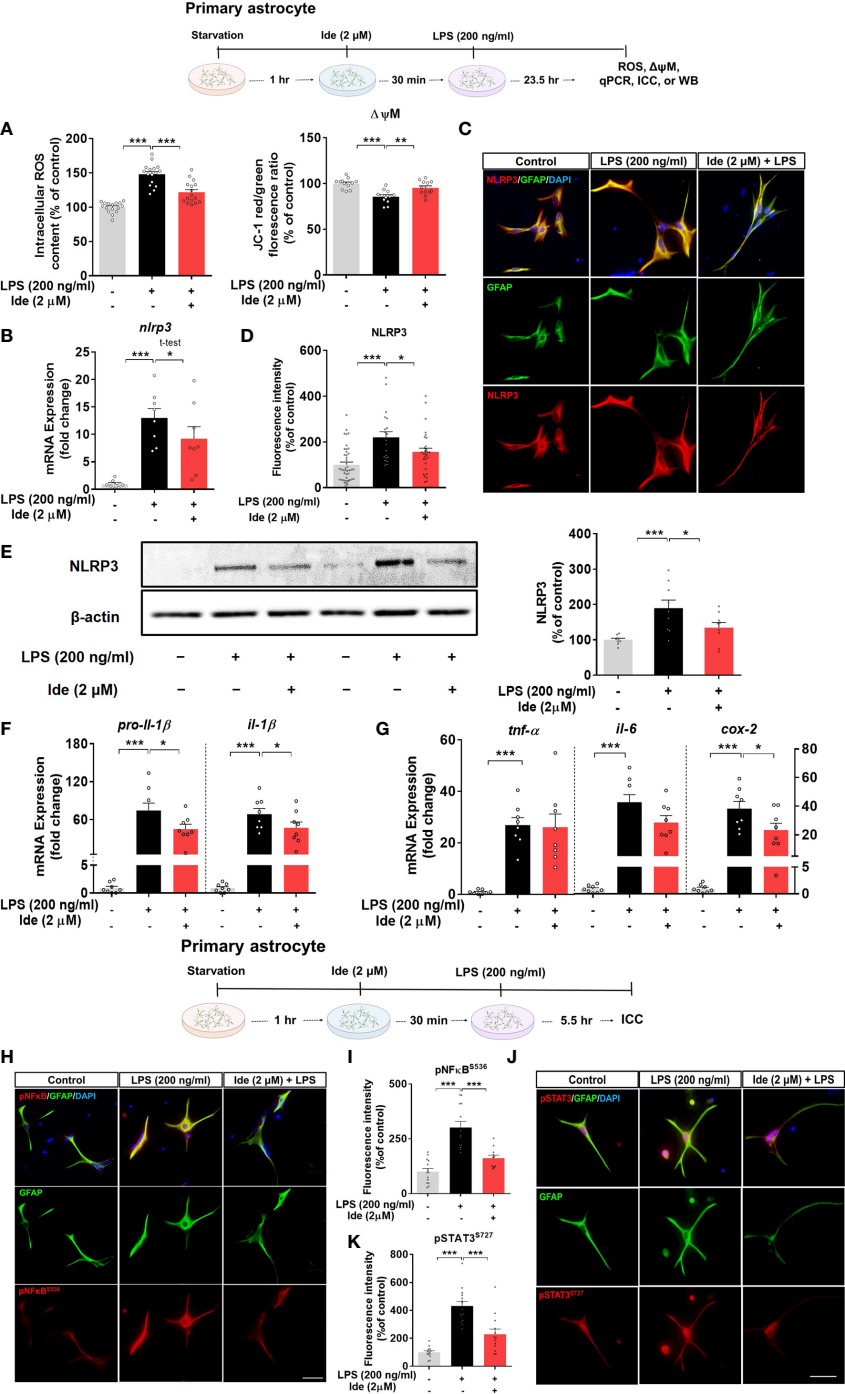
Idebenone suppresses ROS/NLRP3/IL-1β axis activation in LPS-stimulated primary astrocytes. Cells pretreated with idebenone (2 μM) or vehicle (1% DMSO) for 30 min were treated with LPS (200 ng/ml) or PBS for 23.5 h. ROS and mitochondrial membrane potential were analyzed, and real-time PCR and immunocytochemistry were performed. **(A)** Relative intracellular ROS levels and mitochondrial membrane potential (ROS, n = 16/group; mitochondrial membrane potential, n = 12/group). **(B)** Relative *nlrp3* mRNA levels (n = 8/group). **(C, D)** Representative immunocytochemistry image and quantification of NLRP3 expression (Veh, n = 44; LPS, n = 35; and Ide + LPS, n = 41). **(E)** Representative immunoblots and quantification of NLRP3 protein levels (n = 9/group). **(F)** Relative mRNA levels of *pro-il-1β* and *il-1β* (n = 8/group). **(G)** Relative mRNA levels of *tnf-α, il-6*, and *cox-2* (n = 8/group). **(H–K)** Immunocytochemistry of p-NF-kB^S536^ and p-STAT3^S727^ levels in primary astrocytes (Veh, n = 14; LPS, n = 13; and Ide + LPS, n = 14 for p-NF-kB^S536^ and Veh, n = 14; LPS, n = 13; and Ide + LPS, n = 13 for p-STAT3^S727^). *p < 0.05, **p < 0.01, and ***p < 0.001. Scale bar = 50 μm.

Since we observed that idebenone affected LPS-induced neuroinflammation *via* NLRP3 inflammasome signaling in BV2 microglial cells, we further examined whether idebenone alters LPS-evoked ROS/NLRP3/IL-1β axis activation in primary astrocytes. ROS and mitochondrial function were measured in primary astrocytes treated with 2 μM idebenone or 1% DMSO for 30 min followed by 200 ng/ml LPS or PBS for 23.5 h. Idebenone significantly reversed the increase in ROS levels and disruption of mitochondrial membrane potential induced by treatment with LPS in primary astrocytes (**Figure 8A**). Moreover, idebenone reduced LPS-mediated NLRP3 levels and *pro-il-1β* and *il-1β* mRNA levels in primary astrocytes (**Figures 8B–F**). Among proinflammatory cytokines, idebenone reduced LPS-evoked *cox-2* mRNA levels but not *tnf-α* and *il-6* mRNA levels in primary astrocytes (**Figure 8G**).

To identify the changes in transcription factors in the nucleus underlying the modulation of LPS-induced NLRP3-proinflammatory responses by idebenone, ICC was conducted with anti-GFAP and anti-p-STAT3^S727^ or anti-GFAP and anti-p-NF-kB^S536^ antibodies. Idebenone significantly reduced LPS-induced nuclear p-NF-kB and p-STAT3 levels in primary astrocytes (**Figures 8H–K**), suggesting that idebenone modulates LPS-induced NF-kB/STAT3 signaling in primary astrocytes.

### Idebenone Improves Short-Term and Recognition Memory in 5xFAD Mice

**Figure 9:**
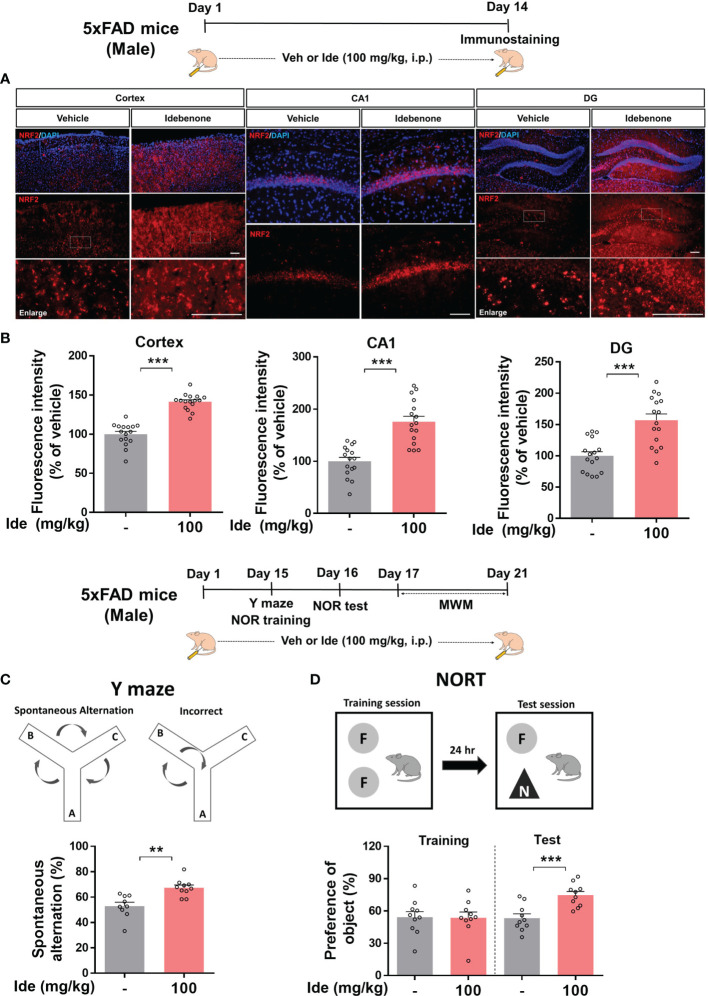
Idebenone increases levels of the neuroprotective marker NRF2 and ameliorates Aβ-mediated cognitive dysfunction in 5xFAD mice. **(A)** Three-month-old 5xFAD mice were administered idebenone (100 mg/kg, i.p.) or vehicle (5% DMSO + 10% PEG + 20% Tween80) daily for two weeks. Then, immunofluorescence staining with an anti-NRF2 antibody or behavioral tests were performed. **(B)** Quantification of NRF2 immunofluorescence intensity (n = 16 brain slices from 4 mice/group). **(C)** Y maze diagram and quantification of spontaneous alternation (n = 9-10/group). **(D)** NOR test diagram and quantification of object preference (n = 9-10/group). **p < 0.01 and ***p < 0.001. Scale bar = 100 μm.

Given the neuroprotective effects of idebenone in LPS-treated WT mice, we turned our attention to the neuroprotective properties of idebenone in 5xFAD mice. 5xFAD mice were injected with 100 mg/kg idebenone (i.p.) or vehicle daily for 2 weeks, and brain sections were immunostained with an anti-NRF2 antibody. Idebenone significantly increased NRF2 levels in the cortex and hippocampus in 5xFAD mice compared with vehicle-treated 5xFAD mice (**Figures 9A, B**).

We then examined whether idebenone affects cognitive function in this mouse model of AD by conducting Y maze, NOR, and Morris water maze tests. Interestingly, idebenone-injected 5xFAD mice exhibited significant enhancement of spontaneous alternations in the Y maze test, implying that idebenone improved the short-term spatial memory impairment associated with Aβ (**Figure 9C**). In addition, idebenone significantly increased recognition memory (**Figure 9D**) but not long-term spatial memory in 5xFAD mice (**Supplementary Figure 3**). These data indicate that idebenone improves cognitive function in this mouse model of AD.

### Idebenone Downregulates Aβ-Associated Microglial and Astrocytic Activation in 5xFAD Mice

**Figure 10:**
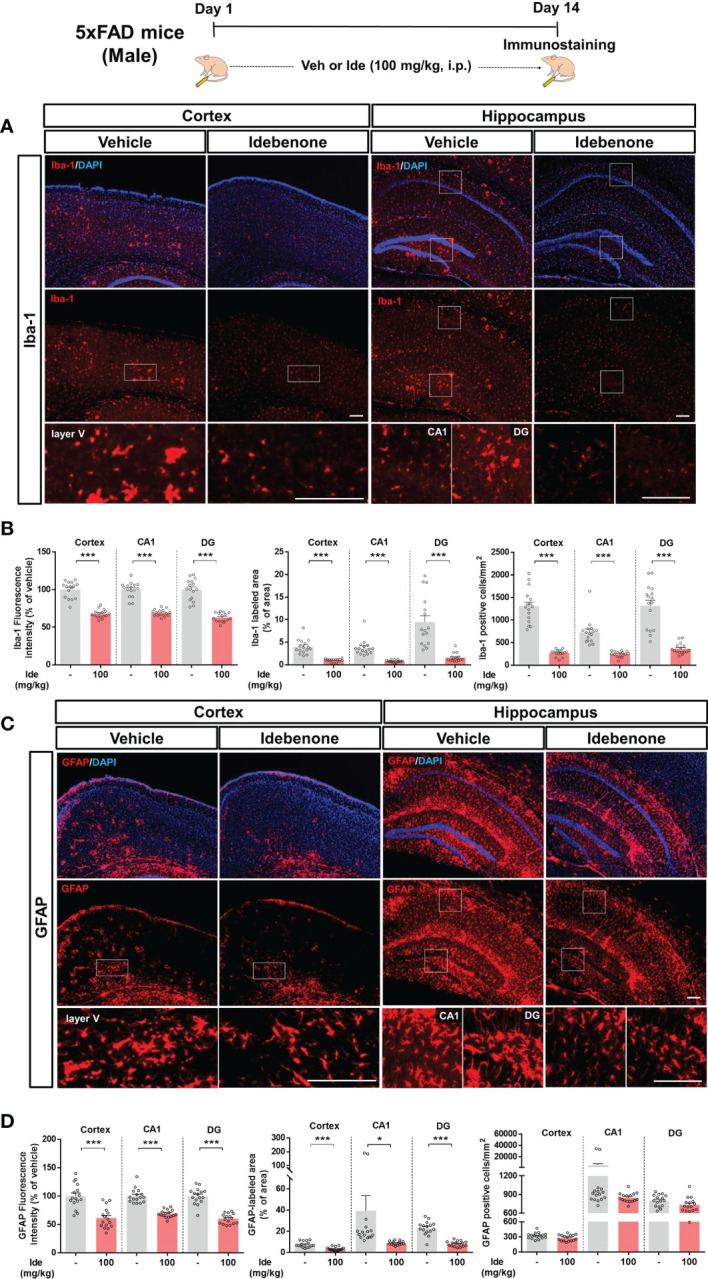
Idebenone attenuates Aβ-associated micro/astrogliosis and micro/astroglial hyperplasia and proliferation in 5xFAD mice. **(A, C)** Representative images of Iba-1 and GFAP immunofluorescence in the cortex and hippocampus. Idebenone (100 mg/kg, i.p.) or vehicle was administered to 3-month-old 5xFAD mice daily for 14 consecutive days. After the last injection, the mice were sacrificed, and brain sections were immunostained with anti-Iba-1 or anti-GFAP antibodies. **(B)** Quantification of the fluorescence intensity of Iba-1; Iba-1-immunolabeled area; and number of Iba-1-positive cells (n = 16 brain slices from 4 mice/group). **(D)** Quantification of the fluorescence intensity of GFAP; GFAP-labeled area; and number of GFAP-positive cells (n = 16 brain slices from 4 mice/group). *p < 0.05 and ***p < 0.001. Scale bar = 200 μm.

Aberrant microglial and astrocytic activation play crucial roles in the progression of AD pathophysiology (31). Therefore, we examined whether idebenone modulates Aβ-associated gliosis in a mouse model of AD. For this experiment, 3-month-old 5xFAD mice were administered 100 mg/kg idebenone (i.p.) or vehicle (5% DMSO + 10% PEG + 20% Tween80) daily for 14 consecutive days, followed by IF staining with anti-Iba-1 or anti-GFAP antibodies. Idebenone treatment significantly reduced Iba-1 intensity, Iba-1-labeled area, and the number of Iba-1-positive microglia in the cortex and hippocampus in 5xFAD mice (**Figures 10A, B**). In addition, idebenone significantly diminished GFAP fluorescence intensity and GFAP-immunopositive area in the cortex and hippocampus in 5xFAD mice, whereas the number of GFAP-positive astrocytes did not change (**Figures 10C, D**). These data indicate that idebenone suppresses microglial/astrocytic activation, microglial/astrocytic hypertrophy, and microglial proliferation in an AD animal model.

### Idebenone Inhibits Aβ-Mediated NLRP3 Inflammasome Activation and Proinflammatory Cytokine Levels in 5xFAD Mice

**Figure 11:**
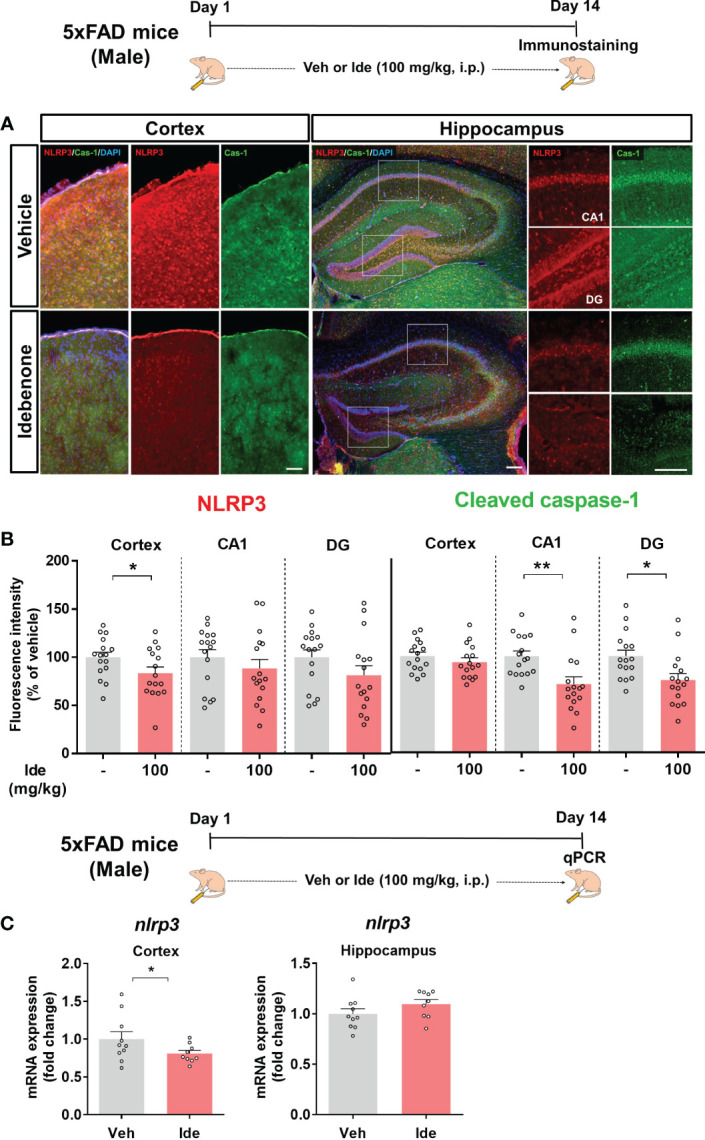
Idebenone inhibits Aβ-mediated NLRP3/caspase-1 axis activation in 5xFAD mice. **(A)** Representative images of NLRP3 and cleaved caspase-1 immunofluorescence in the cortex and hippocampus. **(B)** Quantification of the fluorescence intensity of NLRP3 and cleaved caspase-1 (n = 16 brain slices from 4 mice/group). **(C)** Real-time PCR detection of relative mRNA levels of *nlrp3* in the cortex and hippocampus in 5xFAD mice (n = 9-10 mice/group). *p < 0.05 and **p < 0.01. Scale bar = 200 μm.

Clinical and animal studies have shown that NLRP3 inflammasome activation is associated with the progression of AD pathology (32). Since idebenone modulated LPS-mediated neuroinflammatory responses *via* the NLRP3 inflammasome in WT mice, we investigated whether idebenone affects Aβ-induced NLRP3 inflammasome activation in a mouse model of AD. Three-month-old 5xFAD mice were injected daily with 100 mg/kg idebenone (i.p.) or vehicle (5% DMSO + 10% PEG + 20% Tween80) for 14 days. After 14 days, IF staining was conducted with anti-NLRP3 and anti-cleaved caspase-1 antibodies. Interestingly, NLRP3 immunoreactivity was significantly decreased in the cortex but not the hippocampus in idebenone-treated 5xFAD mice (**Figures 11A, B**). Moreover, idebenone significantly decreased cleaved caspase-1 fluorescence intensity in the hippocampal CA1 and DG region in 5xFAD mice (**Figures 11A, B**). To further confirm our findings, we conducted real-time PCR, which revealed that idebenone significantly downregulated *nlrp3* mRNA levels in the cortex but not the hippocampus in 5xFAD mice treated as described above (**Figure 11C**).

**Figure 12:**
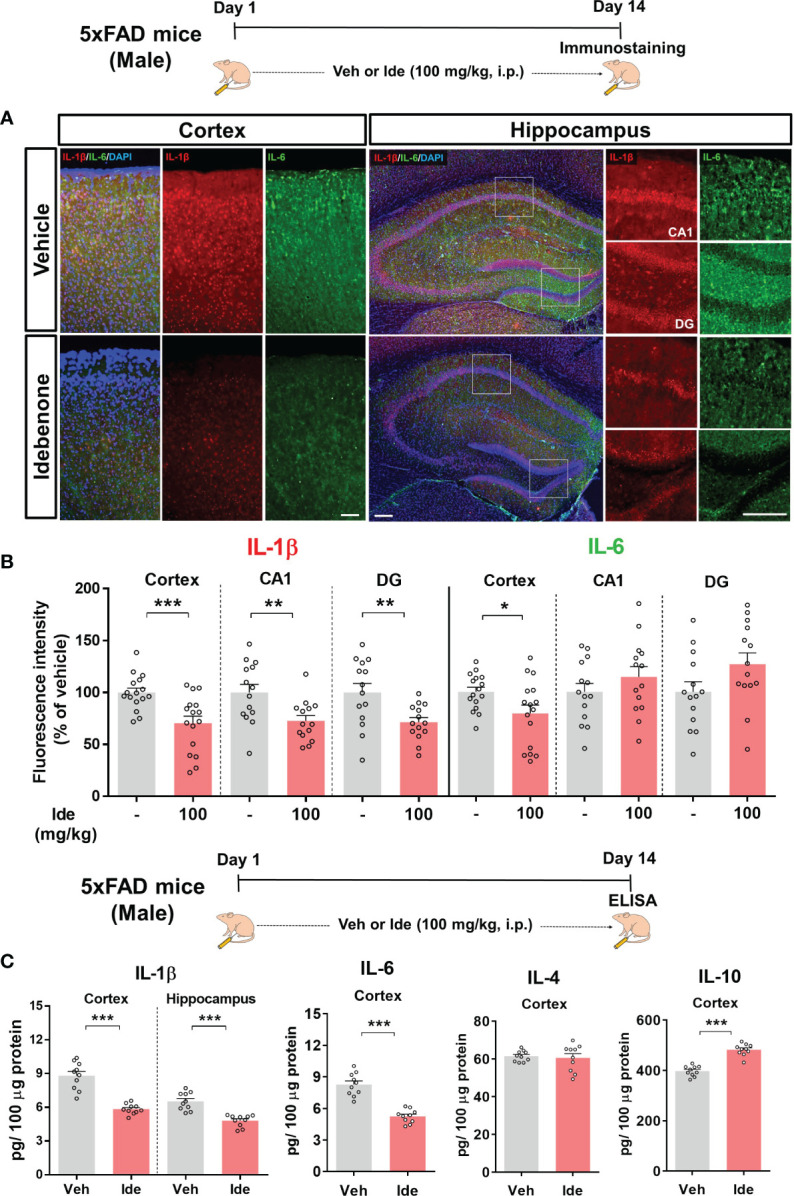
Idebenone modulates levels of the Aβ-evoked proinflammatory cytokines IL-1β and IL-6 in 5xFAD mice **(A)** Representative images of IL-1β and IL-6 immunofluorescence in the cortex and hippocampus in 5xFAD mice. **(B)** Quantification of the fluorescence intensity of IL-1β and IL-6 (n = 14–16 brain slices from 4 mice/group). **(C)** ELISA analysis of IL-1β, IL-6, IL-4, and IL-10 protein levels in the cortex and/or hippocampus in 5xFAD mice (n = 10 mice/group). *p < 0.05, **p < 0.01 and ***p < 0.001. Scale bar = 200 μm.

We then assessed the effect of idebenone on NLRP3 inflammasome-associated proinflammatory cytokine levels in 5xFAD mice by performing IF staining with anti-IL-1β or anti-IL-6 antibodies. In idebenone-injected 5xFAD mice, IL-1β immunoreactivity was significantly decreased in the cortex and hippocampus, whereas IL-6 immunoreactivity was diminished only in the cortex (**Figures 12A, B**). Further analysis by ELISA showed that idebenone treatment significantly decreased cortical/hippocampal IL-1β and cortical IL-6 levels and significantly increased cortical IL-10 levels but not IL-4 levels (**Figure 12C**). These data indicate that idebenone regulates Aβ-mediated neuroinflammatory responses by inhibiting proinflammatory cytokine release in a mouse model of AD.

## Discussion

Sustained neuroinflammation mediated by diseased microglia and reactive astrocytes results in neuronal damage like synaptic loss and, in turn, cognitive dysfunction in neurogenerative diseases such as AD (33, 34). LPS and Aβ are major causes of neuroinflammation and enhance proinflammatory responses by enhancing NLRP3 priming and activating NLRP3 inflammasome assembly, respectively (2, 35, 36). Therefore, in the present study, we used two neuroinflammation-associated disease models (i.e., LPS-injected WT mice and Aβ-expressing 5xFAD mice) to examine whether idebenone differentially regulates LPS-primed NLRP3 and Aβ-activated NLRP3 to modulate subsequent neuroinflammatory responses and cognitive impairments.

Here, we demonstrated that idebenone had neuroprotective effects and attenuated LPS-mediated cognitive dysfunction in male and female WT mice. Moreover, idebenone modulated LPS-associated neurogliosis by inhibiting ROS/NLRP3/IL-1β axis activation in BV2 microglial cells, primary astrocytes, and WT mice. We further found that idebenone contributed to neuroprotection against Aβ and improved learning and memory function in 5xFAD mice. Idebenone-treated 5xFAD mice exhibited reductions in micro/astroglial activation as well as neuroglial hypertrophy and proliferation. Importantly, idebenone ameliorated the vicious NLRP3/caspase-1/IL-1β cycle mediated by Aβ deposition in 5xFAD mice. These data suggest that idebenone is a plausible therapeutic agent for neuroinflammatory and neurodegenerative diseases, including AD.

NRF2 plays a critical role in neuroprotection/anti-inflammation under oxidative stress conditions by translocating into the nucleus to suppress NF-kB signaling *via* modulation of HO-1/bilirubin/Fe^2+^ activity (37, 38). The effects of LPS on cell viability and NLRP3 activity in BV2 microglial cells are dependent on NRF2 (39). Idebenone is an antioxidative agent with therapeutic efficacy for Friedreich’s ataxia (FRDA) based on the upregulation of NRF2 mRNA and protein levels, as assessed in fibroblasts derived from the skin of FRDA patients (40). However, whether idebenone modulates NRF2 activity under LPS- and/or Aβ-stimulated inflammatory circumstances has not been fully investigated. In the present study, IF staining with an anti-NRF2 antibody showed that idebenone remarkably enhanced NRF2 levels in LPS-injected male and female WT mice (**Figure 1**). Taken together with previous reports, our findings suggest that idebenone has neuroprotective effects based on the suppression of central/peripheral inflammatory responses in LPS-injected WT mice.

Previous studies have reported memory-enhancing effects of idebenone in various animal models. For example, idebenone ameliorates Aβ-induced hippocampus-dependent memory impairments in WT mice (41) and improves working and spatial memory deficits in rat models of ischemia and vascular dementia (42, 43). Furthermore, idebenone remarkably rescues short-term memory loss induced by administration of the cholinergic antagonist scopolamine or serotonin deficiency in rats (44). However, these studies have not established whether idebenone improves cognitive deficits evoked by persistent inflammatory stimulation. In this study, cognitive behavioral tests revealed that idebenone attenuated neuroinflammation-mediated short and long-term memory impairments induced by repeated LPS stimulation in male and female WT mice (**Figure 2**). Previous reports have indicated that male WT mice were more susceptible in response to LPS stimulation (i.e., sickness behavior, learning and memory deficits) than female WT mice (45, 46). However, no sex differences in LPS-evoked memory impairment or the memory-improving effects of idebenone were observed in WT mice in the present study (**Figure 2**). Given that neuronal synaptic interconnections are responsible for memory formation and storage, enhancement of neuroprotection implies improvement of cognitive ability (47, 48). Therefore, the improvements in recognition and spatial memory function in LPS-administered mice treated with idebenone are likely due to neuroprotective effects of idebenone against LPS stimulation. Further study will reveal whether idebenone modulates dendritic spine formation and/or LTP (long-term potentiation) to enhance cognitive function.

Excessive glial activation is the predominant consequence of LPS administration (49), and aberrant neuroinflammation contributes to memory impairment (23, 24), giving rise to the hypothesis that idebenone downregulates LPS-induced neurogliosis and inflammatory responses to ameliorate the memory deficits evoked by LPS. Idebenone has been reported to decrease inflammatory microglial activity in MPTP-induced Parkinsonian mice and retinal astrogliosis in a mouse model of Leber’s hereditary optic neuropathy (LHON) (17, 50). However, the effects of idebenone on LPS-induced gliosis *in vivo* have not been previously evaluated. We found that daily injections of idebenone for 3 consecutive days inhibited LPS-mediated astrocytic but not microglial activation in WT mice (**Supplementary Figure 1**). When the treatment period was increased to 8 consecutive days of daily injections, idebenone significantly suppressed both microgliosis and astrogliosis in LPS-stimulated male and female WT mice (**Figures 3**, **4**). Collectively, these results indicate that idebenone enhances neuroprotection and attenuates neuroglial activation, thereby improving cognitive function in an animal model of neurodegenerative disease.

Aberrant neuroglial excitability evoked by LPS stimulation amplifies NLRP3/caspase-1 inflammasome activation (51, 52). Several studies have demonstrated that idebenone regulates NLRP3 inflammasome activity in rats with occlusion-induced ischemia and in mice with atherosclerosis mediated by apolipoprotein E deficiency (19, 53). Here, we provide interesting evidence that idebenone reduces LPS-evoked hyperactivity of the NLRP3/IL-1β axis and subsequent proinflammatory responses in male and female WT mice (**Figure 5**). Interestingly, idebenone reduced the vicious cycle of NLRP3 inflammasome signaling more effectively in the hippocampus than the cortex in LPS-injected male WT mice (**Figure 5A**), whereas both cortical and hippocampal *nlrp3* mRNA levels were significantly reduced in idebenone-treated female WT mice (**Figure 5D**). These data suggest that idebenone differentially regulates the LPS-induced vicious cycle of NLRP3-mediated inflammation depending on brain region and sex. The brain region-specific differences observed in male WT mice might be due to differences in the mitochondrial response to brain stimulation between the cortex and hippocampus. For instance, in rats, traumatic brain injury (TBI) disrupts mitochondrial morphology in the cortex but not the hippocampus and alters mitochondrial kinetics in the hippocampus but not the cortex (54). The effects of antipsychotic drugs on the activities of enzymes involved in the mitochondrial electron transport chain also differ between the cortex and hippocampus (55). The literature and our findings suggest that the effects of idebenone treatment on mitochondrial dynamics vary between the cortex and hippocampus, giving rise to differences in inflammatory responses in these regions of the brain in LPS-stimulated WT mice. With respect to the differential effects of idebenone on LPS-evoked NLRP3 priming depending on sex, it is possible that sex hormones affect NLRP3 inflammasome activity. In a mouse model of atherosclerosis, testosterone suppresses the NLRP3 inflammasome and inflammation, whereas female sex hormones contribute to NLRP3-induced proinflammatory responses (56). These previous findings suggest that female sex hormones might exaggerate LPS-induced NLRP3 priming, thus resulting in a significant enhancement of cortical *nlrp3* mRNA levels by LPS stimulation only in female WT mice.

ROS acts upstream of NLRP3 and activates NLRP3 activity directly or indirectly *via* MAPK-NF-kB signaling upon LPS binding to TLR4 or sensing of pathogen-associated molecular patterns (12, 57). Idebenone downregulates ROS production induced by oxygen or glucose deprivation in BV2 microglial cells, elevated ROS levels in LHON fibroblasts, and bleomycin-mediated increased ROS levels in myofibroblasts (19, 58, 59). Furthermore, idebenone enhances mitochondrial membrane potential in BV2 microglial cells (19). Based on these previous findings, we hypothesized that idebenone promotes mitochondrial function, thereby decreasing ROS production and suppressing NLRP3 inflammasome activation to reduce LPS-evoked pathogenesis. We found that LPS-evoked intracellular ROS production and disruption of mitochondrial membrane potential were reversed in idebenone-treated BV2 microglial cells and primary astrocytes (**Figures 6**, **8**). Interestingly, idebenone inhibited the LPS-evoked increases in nuclear p-NF-kB and p-STAT3, signaling molecules that act upstream of NLRP3, in both BV2 microglia and primary astrocytes (**Figures 7**, **8**). These data indicate that idebenone regulates LPS-stimulated NLRP3 inflammasome activation by suppressing ROS production and NF-kB/STAT3 signaling.

Our *in vivo* and *in vitro* experiments demonstrated that the effects of idebenone on LPS-mediated NLRP3 inflammatory responses were distinct between microglia and primary astrocytes. There are several possible explanations for these differences. First, differences in NLRP3 expression among cell types could influence the effects of idebenone on Aβ- or LPS-mediated inflammation. NLRP3 is abundantly expressed in microglia and triggers the secretion of proinflammatory mediators and astrocyte activation upon immune stimulation (60–63). We and others have observed NLRP3 expression in primary astrocytes (64–66), but another study reported that mouse astrocytes do not express NLRP3 nor release IL-1β (67). Based on previous work and our current findings, it is likely that NLRP3 is expressed predominantly in microglial cells, and thus the effects of idebenone might be greater in microglial cells than in primary astrocytes. Second, the effects of idebenone on immune responses may be influenced by cell type-dependent expression of effectors that regulate idebenone activity. For instance, expression of NAD(P)H quinone oxidoreductase 1 (NQO1), an enzyme that plays a critical role in the donation of electrons from idebenone in mitochondrial Complex I to Complex III (68), is rarely observed in cortical neurons but is abundant in cortical astrocytes; as a result, the effects of idebenone on mitochondrial function are distinct between astrocytes and neurons (68). Therefore, we speculate that differential expression of effectors of idebenone in microglia and astrocytes underlies the cell type-dependent modulation of the NLRP3/IL-1β axis by idebenone. Further studies are needed to demonstrate whether NQO1 also influences the effects of idebenone on the LPS-induced vicious NLRP3 neuroinflammation cycle.

According to a recent study, coenzyme Q10 administration ameliorates the inflammatory response and enhances mitochondrial function by suppressing microglial activation in Aβ-treated WT mice (69). In addition, coenzyme Q10-injected AβPP/PS1 mice exhibit significantly lower brain levels of protein carbonyls, a product of amplified ROS predominantly produced in mitochondria (70). Although idebenone, a ubiquinone analogue, has neuroprotective effects in Aβ-treated rat hippocampal cultures and memory-enhancing effects in Aβ-treated WT mice (16, 41), the effects of idebenone on Aβ-associated neurogliosis or NLRP3 activation have not been fully investigated in a mouse model of AD. Consistent with these previous reports, we demonstrated that idebenone has neuroprotective effects and enhances short-term and recognition memory in 5xFAD mice (**Figure 9**). Moreover, in the present study, we found that idebenone attenuated Aβ-associated microglial and astrocytic activation in 3-month-old 5xFAD mice (early phase of AD pathology) (**Figure 10**). Our study is also the first to demonstrate that idebenone ameliorates the vicious NLRP3/caspase-1/IL-1β cycle, which contributes to the pathogenesis of familial AD and sporadic AD (32, 71), in 5xFAD mice (**Figures 11**, **12**). Overall, our new findings imply that idebenone regulates the progression of AD pathogenesis by attenuating neurogliosis and NLRP3/IL-1β axis activation. However, it is possible that idebenone regulates other neuroinflammation-related signaling pathways in 5xFAD mice, and our future work will examine whether idebenone alters pathways besides NLRP3 to attenuate Aβ-amplified neuroinflammation.

Idebenone enhanced short-term, recognition, and long-term spatial memory function in LPS-treated WT mice (**Figure 2**) but only short-term and recognition memory in 5xFAD mice (**Figure 9**). Why did idebenone have no effect on long-term spatial memory in 3-month-old 5xFAD mice? There are a few possible explanations for this finding. First, idebenone more effectively regulated LPS-induced NLRP3 priming than Aβ-stimulated NLRP3 activation, and these differential effects might influence the modulation of memory function. As a result, idebenone improved cognitive ability as assessed by the Morris water maze test in LPS-treated WT mice but not Aβ-overexpressing 5xFAD mice. Second, 3-month-old 5xFAD mice are a model of the early phase of AD and have not yet developed impairments of long-term cognitive ability. Spatial working memory impairment is observed in 3-month-old 5xFAD mice (72, 73), but long-term spatial memory deficits are observed only in the intermediate/late phase of AD in this model, i.e., 5xFAD mice older than 6 months (74, 75). Thus, future work will investigate whether idebenone improves long-term memory in 6-month-old 5xFAD mice *via* the Morris water maze test. Third, a treatment period of 2 weeks may not be sufficient to improve long-term memory function in 5xFAD mice. The ability of longer treatment times to effectively increase long-term spatial memory in 5xFAD mice will be evaluated in a future study.

## Conclusion

**Figure 13:**
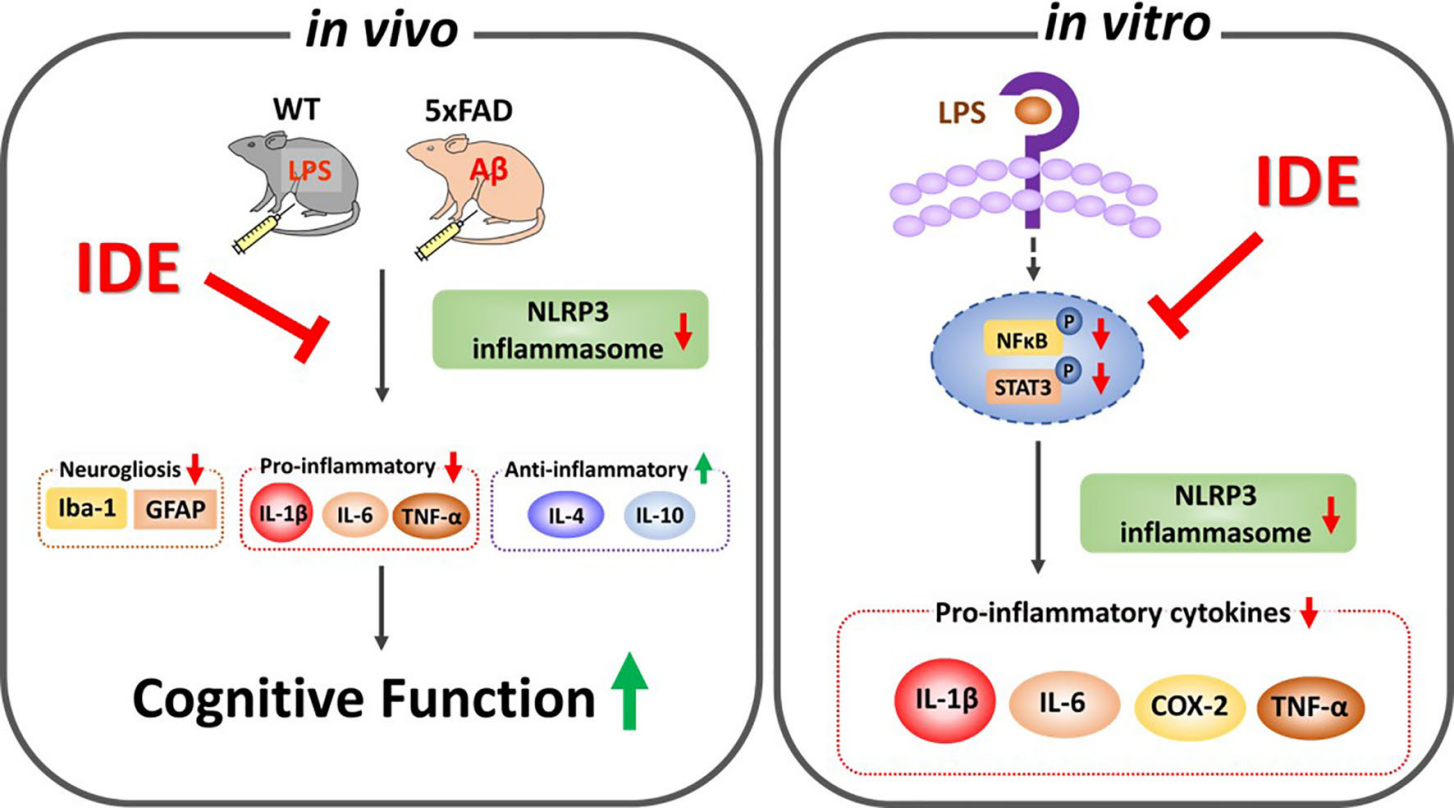
Graphical illustration of the effects of idebenone on LPS- and Aβ-induced neurogliosis and memory impairments *via* suppression of NLRP3-IL-1β axis. *In vitro*, idebenone downregulated LPS-evoked proinflammatory cytokine levels by inhibiting NLRP3 inflammasome. *In vivo*, idebenone decreased neurogliosis and proinflammatory cytokine levels by altering NLRP3 priming/activation thereby improving cognitive function in LPS-treated wild-type mice and 5xFAD mice.

The present study demonstrated that idebenone improves neuroprotection and rescues cognitive deficits induced by sustained immune stimulation, LPS-mediated neurogliosis, NLRP3 inflammasome activation and subsequent inflammatory responses in male and female WT mice. In BV2 microglial cells and primary astrocytes, idebenone modulates the LPS-induced vicious ROS/NLRP3/IL-1β cycle and proinflammatory cytokine levels. In 5xFAD mice, idebenone protects neurons against Aβ and suppresses Aβ-triggered neurogliosis, glial hypertrophy, and glial proliferation by modulating NLRP3/caspase-1/IL-1β signaling. Collectively, our results suggest that idebenone is a plausible therapeutic molecule for targeting neuroglial NLRP3 inflammasome activation and, in turn, inhibiting the pathological progression of neuroinflammation-related diseases, including AD (**Figure 13**).

## Data Availability Statement

The original contributions presented in the study are included in the article/**Supplementary Material**. Further inquiries can be directed to the corresponding author.

## Ethics Statement

All experimental procedures were performed in accordance with the animal protocols and guidelines reviewed and approved by the Korea Brain Research Institute Animal Care and Use Committee (approval no. IACUC-19-00049).

## Author Contributions

H-SH and H-jL conceived and participated in the design of the study, wrote the manuscript, performed experiments, and confirmed all data analyses in all figures and supporting information. J-HP participated in data analysis during the revision. All authors read and approved the final manuscript.

## Funding

This work was supported by the KBRI basic research program through KBRI funded by the Ministry of Science, ICT & Future Planning (grant numbers 21-BR-02-11 and 21-BR-02-22, H-SH) and the National Research Foundation of Korea (grant number 2019R1A2B5B01070108, H-SH).

## Conflict of Interest

The authors declare that the research was conducted in the absence of any commercial or financial relationships that could be construed as a potential conflict of interest.

## Publisher’s Note

All claims expressed in this article are solely those of the authors and do not necessarily represent those of their affiliated organizations, or those of the publisher, the editors and the reviewers. Any product that may be evaluated in this article, or claim that may be made by its manufacturer, is not guaranteed or endorsed by the publisher.
