# Supplementary material for: Idebenone Regulates Aβ and LPS-Induced Neurogliosis and Cognitive Function Through Inhibition of NLRP3 Inflammasome/IL-1β Axis Activation
Source: Front Immunol. 2022 Feb 10;13:749336. doi: 10.3389/fimmu.2022.749336 (PMC8866241; doi:10.3389/fimmu.2022.749336)
Supplement: Supplementary file 1 [file DataSheet_1.docx]

**Supplementary information**

**Idebenone regulates** **Aβ and LPS-induced neurogliosis and cognitive function through inhibition of NLRP3 inflammasome/IL-1β axis activation**

Hyun-ju Lee^1^ | Jin-Hee Park^1^ | Hyang-Sook Hoe^1,2,*^

^1^Department of Neural Development and Disease, Korea Brain Research Institute (KBRI), 61, Cheomdan-ro, Daegu, Republic of Korea; ^2^Department of Brain and Cognitive Sciences, Daegu Gyeongbuk Institute of Science & Technology, Daegu 42988, Korea

*Corresponding author

Hyang-Sook Hoe, Ph.D.: Department of Neural Development and Disease, Korea Brain Research Institute (KBRI), 61 Cheomdan-ro, Dong-gu, Daegu, Korea, 41068

E-mail: [sookhoe72@kbri.re.kr](mailto:sookhoe72@kbri.re.kr)

**
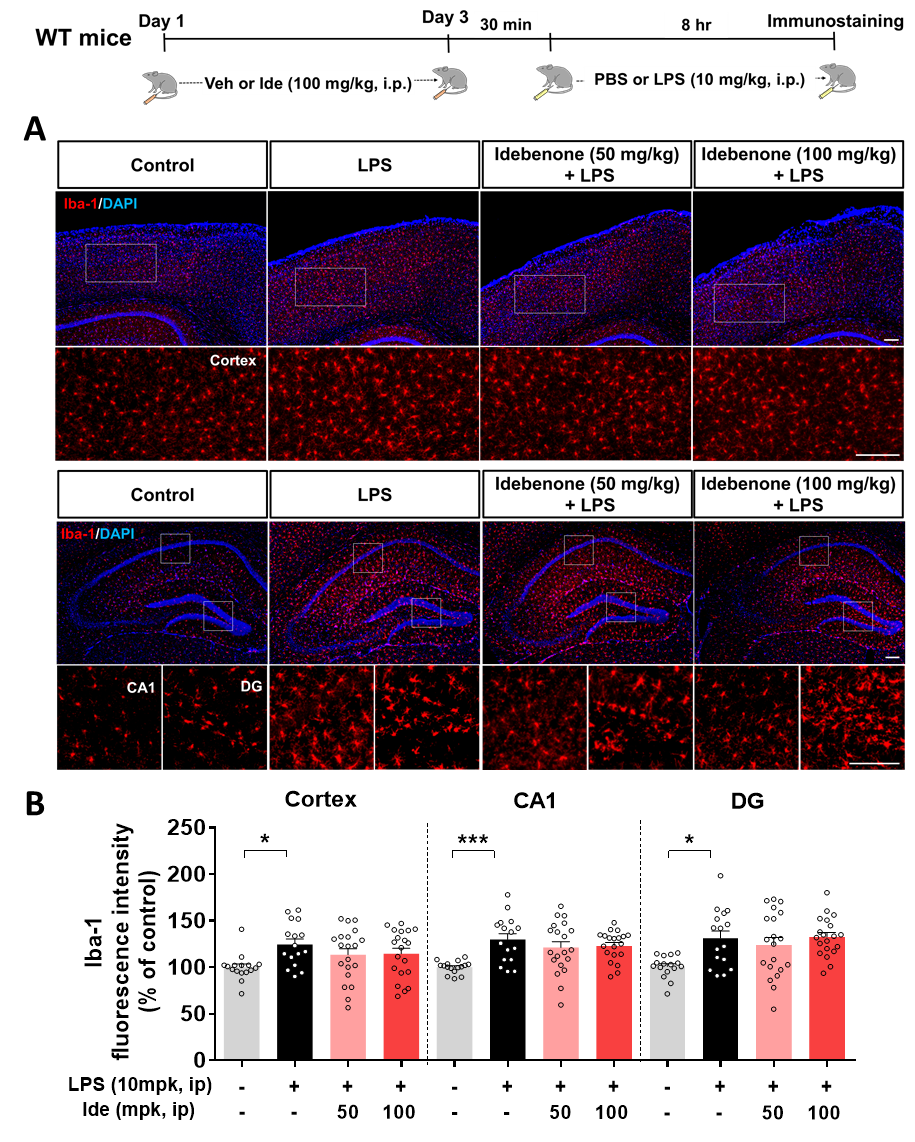
**

**Supplementary Figure 1.** Daily injection of idebenone for 3 consecutive days does not alter microglial activation in LPS-injected 3-month-old male wild-type mice. (**A)** Representative images of Iba-1 immunofluorescence in the cortex and hippocampus. Idebenone (50 or 100 mg/kg, i.p.) or vehicle was administered daily to wild-type mice for 3 days, followed on day 3 by LPS (10 mg/kg, i.p.) or PBS injection. Eight hours after LPS or PBS injection, the mice were sacrificed, and brain tissue was immunostained with an anti-Iba-1 antibody. (**B)** Quantification of Iba-1 fluorescence intensity (n = 16–20 brain slices from 4–5 mice/group). *p < 0.05 and ***p < 0.001 vs. vehicle-treated control. Scale bar = 200 μm.

**
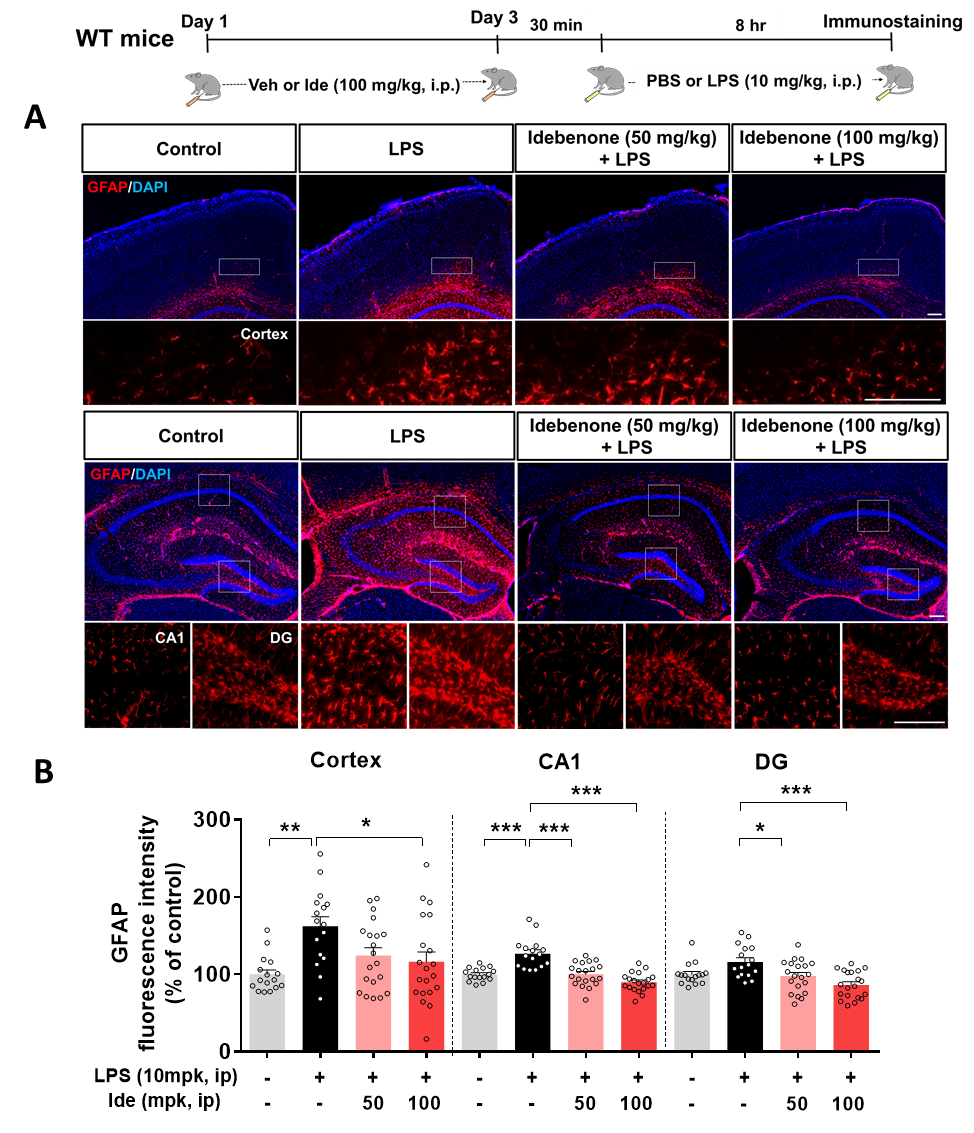
**

**Supplementary Figure 2.** Daily injection of idebenone for 3 consecutive days suppresses astrogliosis in LPS-injected 3-month-old male wild-type mice. (**A**) Representative images of GFAP immunofluorescence in the cortex and hippocampus. Idebenone (50 or 100 mg/kg, i.p.) or vehicle was administered to wild-type mice daily for 3 consecutive days, followed by LPS (10 mg/kg, i.p.) or PBS injection on day 3. Eight hours after LPS or PBS injection, the mice were sacrificed, and brain tissue was immunostained with an anti-GFAP antibody. (**B)** Quantification of fluorescence intensity of GFAP (n = 16–20 brain slices from 4–5 mice/group). *p < 0.05 and ***p < 0.001 vs. vehicle-treated control. Scale bar = 200 μm.

**
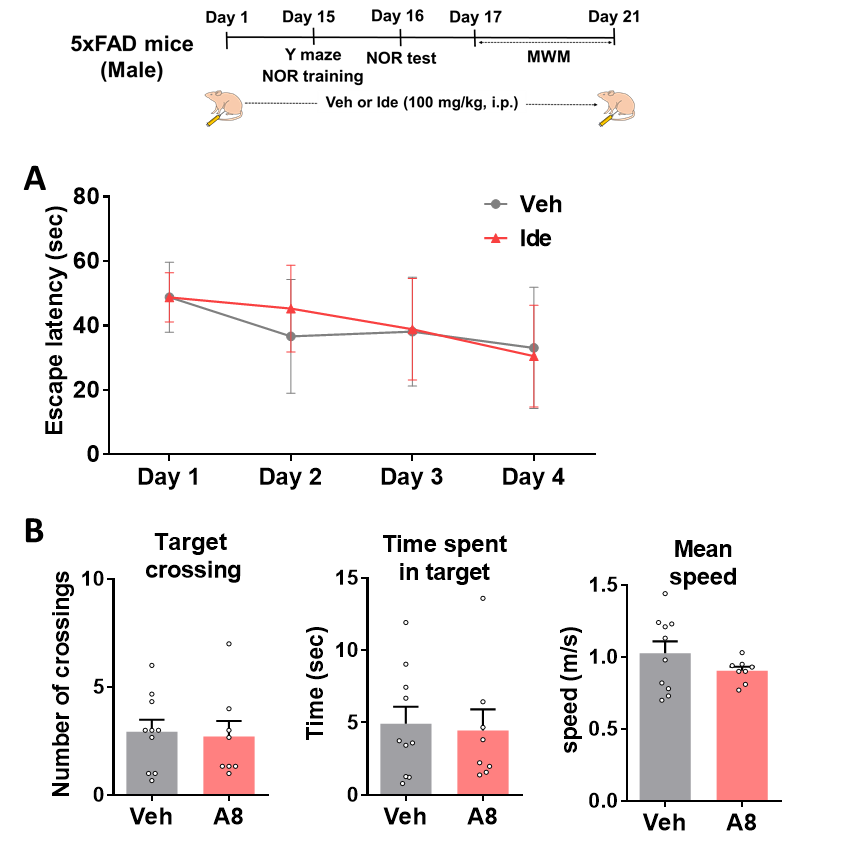
**

**Supplementary Figure 3.** Idebenone does not alter spatial memory in 3-month-old male 5xFAD mice. (**A**) Escape latencies during the training session of the Morris water maze text (n = 9–10/group). (**B**) Time spent in the target quadrant, number of target crossings, and mean speed during probe trial of the Morris water maze test (n = 9–10/group).
